# Gene Therapy for Inflammatory Cascade in Intrauterine Injury with Engineered Extracellular Vesicles Hybrid Snail Mucus‐enhanced Adhesive Hydrogels

**DOI:** 10.1002/advs.202410769

**Published:** 2024-10-25

**Authors:** Xiaotong Peng, Tao Wang, Bo Dai, Yiping Zhu, Mei Ji, Pusheng Yang, Jiaxin Zhang, Wenwen Liu, Yaxin Miao, Yonghang Liu, Shuo Wang, Jing Sun

**Affiliations:** ^1^ Department of Gynecology Shanghai Key Laboratory of Maternal Fetal Medicine Shanghai Institute of Maternal‐Fetal Medicine and Gynecologic Oncology Shanghai First Maternity and Infant Hospital School of Medicine Tongji University Shanghai 200092 China; ^2^ Department of Hematology Huashan Hospital Fudan University Shanghai 200040 China; ^3^ School of Pharmacy and State Key Laboratory of Quality Research in Chinese Medicine Macau University of Science and Technology Macao 999078 China; ^4^ Department of Orthopaedics Shanghai Sixth People's Hospital Affiliated to Shanghai Jiao Tong University School of Medicine Shanghai 200233 China

**Keywords:** adhesive hydrogels, extracellular vesicles, gene therapy, intrauterine adhesions, macrophage polarization

## Abstract

Early hyper‐inflammation caused by intrauterine injury triggered subsequent intrauterine adhesion (IUA). STAT1‐mediated M1 macrophages are confirmed to secrete pro‐inflammatory cytokines to accelerate inflammatory cascade and IUA formation by multi‐omics analysis and experimental verification. However, clinically used hyaluronic acid (HA) hydrogels are prone to slip out of injury sites due to poor bio‐adhesion properties. Therefore, there are still challenges in applying hydrogels for M1 macrophage intervention in IUA treatment. Herein, an engineered extracellular vesicles (EVs) hybrid snail mucus (SM)‐enhanced adhesive hydrogels to improve bio‐adhesion property is fabricated and M1 macrophage intervention through targeting delivery and STAT1 silencing is achieved. First, inspired by the high bio‐adhesion capacity of SM, SM and gelatin methacrylate (GelMA) solution are mixed to construct GelMA/SM (GS) hydrogel. Then, folic acid‐modified extracellular vesicles (FA‐EVs) are synthesized for targeting the delivery of STAT1‐siRNA. Upon injection of FA‐EVs hybrid GS hydrogel into the uterine cavity, a protective hydrogel layer forms on the surface of injury sites and sustains the release of STAT1‐siRNA‐loaded FA‐EVs to curtail M1 macrophages generation through inhibiting STAT1 phosphorylation, resulting in reduction of myofibroblasts activation and collagen deposition. In addition, the pregnancy rate and the number of fetuses in rats treated with this hydrogel were much higher than those in other groups, suggesting that the hydrogel could promote functional endometrial regeneration and restore fertility. Overall, this study presents a promising strategy for employing FA‐EVs hybrid adhesive hydrogel with superior bio‐adhesion properties and M1 macrophage targeting delivery for IUA treatment and uterus recovery.

## Graphical abstract

This study develops a snail mucus‐inspired adhesive hydrogel of siRNA@FA‐EVs/GS for IUA prevention. Upon injection of this hydrogel into the uterine cavity, a protective hydrogel layer forms on the surface of injury sites and sustains the release of STAT1‐siRNA‐loaded FA‐EVs to curtail M1 macrophages polarization through inhibiting STAT1 phosphorylation, resulting in the reduction of myofibroblasts activation and collagen deposition for IUA treatment and uterus recovery.

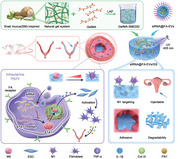

## 1 Introduction

Severe trauma or infection in the basal layer of the endometrium frequently induces excessive inflammation and results in the formation of intrauterine adhesion (IUA) characterized by partial or complete occlusion of the uterine cavity.^[^ ^1^ ^]^ With the increasing number of uterine surgeries, especially post‐miscarriage curettage, the incidence rate of IUA is increasing year by year and causes hypomenorrhea, repeated abortions, and infertility, which seriously endangers women's physical and mental health.^[^ ^2^ ^]^ Currently, the main clinical treatment for IUA is transcervical resection of adhesion (TCRA),^[^ ^3^ ^]^ but new trauma from secondary surgery may trigger adhesion reformation and the recurrence rate of postoperative re‐adhesion after surgery is reportedly as high as 62.5%.^[^ ^4^ ^]^ Post‐treatment strategies primarily involve injecting physical barriers such as balloon uterine stent,^[^ ^5^ ^]^ Foley catheters,^[^ ^6^ ^]^ and hyaluronic acid (HA) gel^[^ ^7^ ^]^ to restore the patency of the uterine cavity or cover injury sites for preventing adhesion invasion. Unfortunately, the shape of solid devices like stents or catheters is unable to be free modified for personalized customization and often causes sterile inflammation and pain after implantation.^[^ ^8^ ^]^ In addition, the bio‐adhesion of HA gel is poor, which indicates that HA gel is prone to slip out of injury sites and results in anti‐adhesion efficacy reduction.^[^ ^9^ ^]^ Therefore, there is an urgent need to fabricate anti‐adhesion biomaterials to precisely inhibit inflammation and persistently adhere to the injury sites.

As the key regulator in post‐trauma inflammation, macrophages and their released cytokines are critical to inflammatory infiltration and hyperplasia and recognized to be the “initiator” of IUA.^[^ ^10^ ^]^ By virtue of their high degree of heterogeneity and plasticity, macrophages can polarize to different subtypes in response to various immune microenvironments to exert pro‐inflammatory (classical activation, M1 subtype) or anti‐inflammatory (alternative activation, M2 subtype) effects at different stages of IUA, thereby regulating the repair of the uterine cavity and adhesion formation.^[^ ^11^ ^]^ Previous research demonstrated that inhibition of excessive inflammation could ameliorate IUA.^[^ ^12^ ^]^ Of the two subtypes, M1 macrophages are more strongly associated with early hyperinflammation.^[^ ^13^ ^]^ After an intrauterine injury or infection, macrophages are recruited from peripheral blood and polarize to the M1 subtype to produce pro‐inflammatory cytokines such as tumor necrosis factor‐alpha (TNF‐α) and interleukin‐1β (IL‐1β), which induce the transformation of endometrial stromal cells (ESCs) into myofibroblasts, triggering the excessive collagen deposition and accelerate the progress of IUA.^[^ ^14^ ^]^ However, the underlying mechanism of M1 polarization after intrauterine injury remains unrevealed. Signal transducers and activators of transcription 1 (STAT1) have been widely reported to participate in diverse inflammatory, fibrotic, and adhesion diseases by acting as positive transcription regulators to activate the downstream pathway and contribute to the polarization of M1 macrophage and pro‐inflammatory cytokine release.^[^ ^15^ ^]^ Nevertheless, whether STAT1 is involved in the process of IUA by mediating macrophage M1 polarization has not yet been reported. We speculated that the targeted and precise intervention of STAT1‐mediated macrophage M1 polarization and inflammatory cytokines release may be effective in reducing the occurrence of IUA.

Gene therapy is currently regarded as the promising treatment for complex disorders via the delivery of therapeutic plasmids or small interference RNAs (siRNAs) into diseased cells to reverse the progress of diseases.^[^ ^16^ ^]^ To ensure delivery into target cells and avoid degradation by ribonucleases, the choice of vehicle is particularly critical. With great biocompatibility and low immunogenicity, EVs have been developed as delivery carriers for drugs and siRNA, and have been widely used in the treatment of various diseases.^[^ ^1^, ^17^ ^]^ Whereas, due to a lack of cellular targeting, unmodified EVs present poor target cell uptake efficiency and unexpected side effects caused by erroneous entry into other cells.^[^ ^18^ ^]^ Therefore, constructing M1 macrophage‐targeted EVs to deliver STAT1‐siRNA for precise targeted intervention is crucial for gene therapy of IUA. Folic acid (FA) receptor is a class of glycosyl phosphatidylinositol‐coupled glycoproteins expressed in cell membranes and mainly distributed on the surface of activated M1 macrophages.^[^ ^19^ ^]^ FA and its analogs can specifically bind to FA receptors on the surface of M1 macrophages and help the delivery vector to be phagocytosed.^[^ ^20^ ^]^ Therefore, the FA modification conferred the M1 macrophage‐targeted function to EVs. Then, loading STAT1‐siRNA enables targeted gene therapy for macrophage M1 polarization.

Hydrogel has a porous 3D network structure and can be used as the carrier for controlled‐release EVs to enable the sustained release of siRNA at injury sites.^[^ ^21^ ^]^ Gelatin methacrylate (GelMA) is one of the most commonly used hydrogel materials, and uniformly encapsulates EVs in the 3D network by UV light curing to achieve long‐term controlled release.^[^ ^22^ ^]^ However, previous investigations reported that the adhesion properties of GelMA hydrogel were unsatisfactory.^[^ ^23^ ^]^ Poor adhesion properties are the main reasons for the failure of the hydrogel slips out of injury sites and anti‐adhesion efficacy reduction.^[^ ^24^ ^]^ Therefore, improved hydrogel bio‐adhesion to the surface of the uterine cavity can overcome this limitation. Incorporation of adhesive substances into the hydrogel network is an effective method to give the hydrogel system an excellent ability to adhere to the injured tissue and prevent dislodgement.^[^ ^25^ ^]^ Inspired by the strong bio‐adhesion property of snail mucus (SM) enables snails to crawl on smooth surfaces.^[^ ^9^ ^]^ Combined with the advantages of SM, a novel GelMA‐SM (GS) adhesive hydrogel can be obtained by adding SM into GelMA solution and then UV light curing.

**Scheme 1:**
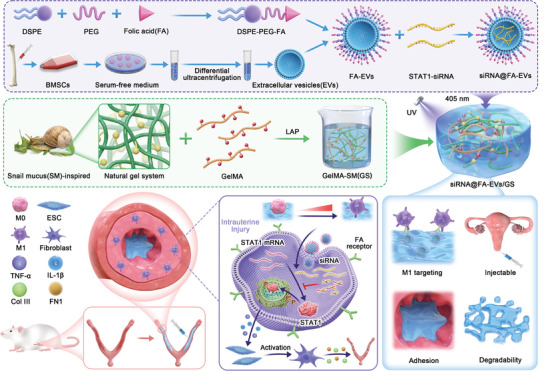
Schematic representation of siRNA@FA‐EVs/GS hydrogel inhibited M1 macrophage polarization and pro‐inflammatory cytokines production through the inhibition of STAT1 phosphorylation, which reduced the transformation of endometrial stromal cells into myofibroblasts and collagen deposition, and inhibited the formation of intrauterine adhesion.

In this study, we demonstrated that STAT1 manipulated endometrial M1 macrophage polarization by multi‐omics analysis and in vivo experiments and reported a novel synergetic delivery platform to achieve gene therapy of IUA and promote uterus recovery through encapsulating STAT1‐siRNA into M1 macrophage‐targeted engineered EVs. Distearoyl phosphoethanolamine‐polyethylene glycol‐FA (DSPE‐PEG‐FA) was prepared and embedded in the surface of the EVs membrane to precisely deliver STAT1‐siRNA into M1 macrophage in the inflammatory phase and then incorporated into snail mucus‐inspired adhesive hydrogel to closely adhere to injury sites and sustained release into inflammation sites. We hypothesized that this EVs‐hydrogel could release M1 macrophage‐targeted EVs to pinpoint target cells and efficiently inhibit STAT1‐mediated inflammatory signaling to suppress macrophage M1 polarization and pro‐inflammatory cytokines release, resulting in alleviating the transformation of ESCs into myofibroblasts and collagen deposition (**Scheme** **1**). In addition, the pregnancy rate and the number of fetuses in rats treated with this hydrogel were much higher than those in other groups, suggesting that the hydrogel could promote functional endometrial regeneration and restore fertility. Overall, our study provides an alternative strategy to achieve gene therapy of IUA and promote uterus recovery for efficient inhibition of macrophage M1 polarization and improved adhesive property of anti‐adhesion hydrogel.

## 2 Results and Discussion

### 2.1 Multi‐omics Analysis and Cell Co‐localization of Phosphorylated STAT1 (pSTAT1)

**Figure 1:**
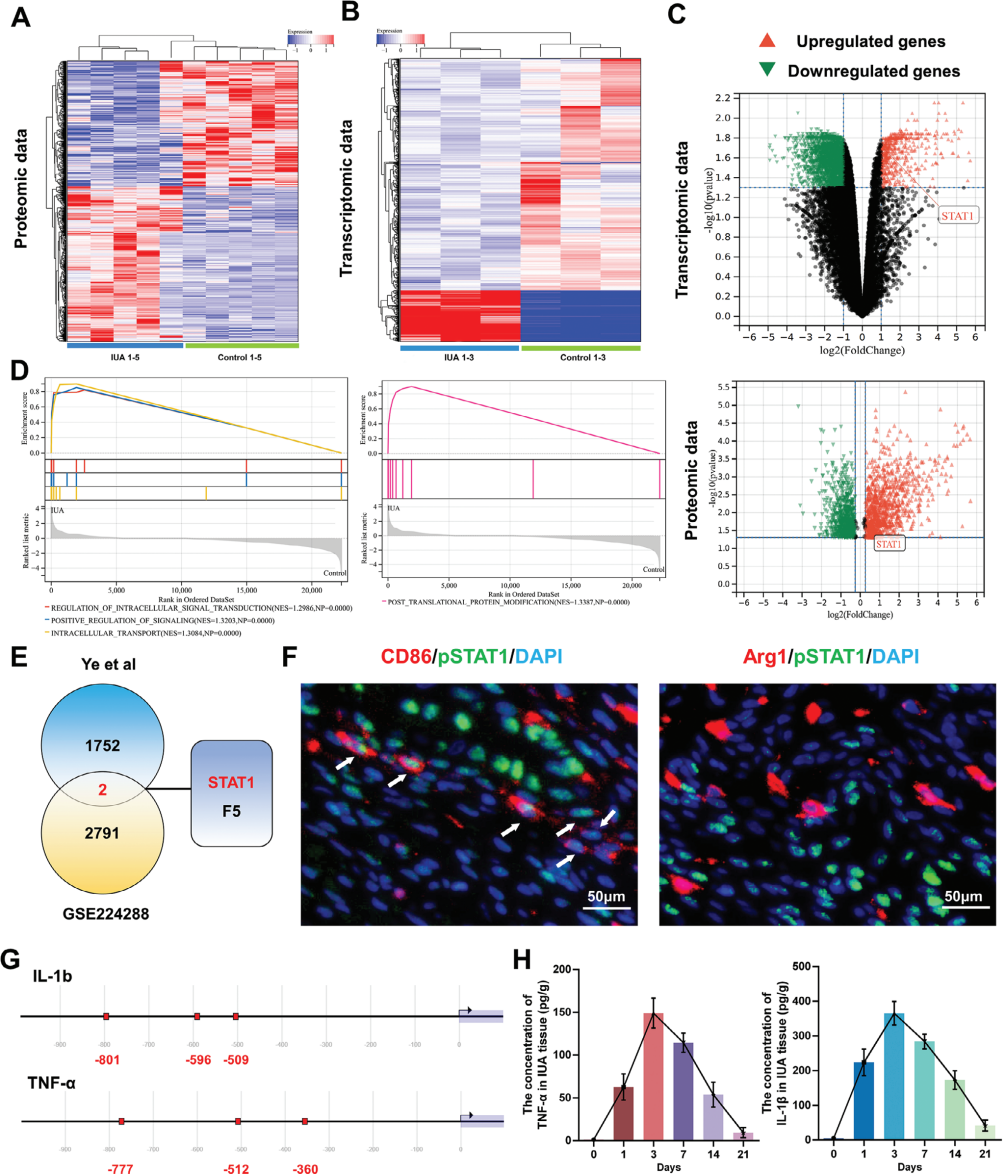
A, B) Heatmap analysis of proteomics and transcriptomic dataset. C) Volcano plot exhibited DEGs and DEPs in the transcriptomic dataset and proteomics dataset. D) Gene set enrichment analysis (GSEA) of genes altered GO and KEGG pathway analysis. E) Venn diagram analysis of the intersection of transcriptomic data and proteomics data. F) Immunofluorescence co‐localization of pSTAT1 in M1 (CD86‐labeled) and M2 (Arg1‐labeled) macrophages in IUA. G) STAT1 binding sites in the promoter region of TNF‐α and IL‐1β. H) The concentration of TNF‐α and IL‐1β in the disease course (0, 1, 3, 7, 14, 28 days after surgery), Data are presented as mean ± SD, *n =* 3.

To explore the key regulatory targets for macrophage‐mediated inflammatory cascade in IUA, we obtained proteomics (Ye et al)^[^ ^26^ ^]^ and microarray (GSE224288) of IUA in public reports and databases, and performed comprehensive multi‐omics analysis to predict differentially expressed proteins (DEPs) and genes (DEGs). 1754 DEPs of IUA tissues and 2793 DEGs of macrophages in IUA tissues were screened and visualized respectively through heatmaps and volcano plots (**Figure** **1**A–C). In addition, we also applied gene set enrichment analysis (GSEA) of the IUA microarray and found that intracellular signal transduction and post‐transcriptional protein modification play a central role in regulating macrophage function after intrauterine injury (Figure 1D). Furthermore, the gene ontology (GO) and Kyoto Encyclopedia of Genes and Genomes (KEGG) analysis of up‐regulated and down‐regulated DEPs and DEGs were also performed (Figures S1–S4, Supporting Information). Through taking the interaction between DEPs and DEGs, from the two datasets, two key genes (STAT1 and F5) were obtained (Figure 1E). The full name of F5 is coagulation factor V and it serves as a critical cofactor for the prothrombinase activity to accelerate the transformation of prothrombin into thrombin. Knockout of this gene results in autosomal recessive hemorrhage or autosomal dominant thrombosis.^[^ ^27^ ^]^ In contrast, STAT1 has been widely reported in a variety of inflammatory diseases and identified as a key transcription factor regulating macrophage M1 polarization.^[^ ^28^ ^]^ STAT1 is able to activate downstream pathways through autophosphorylation, thereby modulating inflammatory response and fibrosis formation. Yang et al. demonstrated that inhibition of STAT1 could reduce liver fibrosis and inflammation in mice by suppressing macrophage M1 polarization.^[^ ^29^ ^]^ Zhao et al. effectively reduced abdominal adhesions by modulating STAT1 pathway‐mediated macrophage polarization.^[^ ^30^ ^]^ However, the role of STAT1 in mediating macrophage polarization and immune microenvironment after intrauterine injury has not yet been revealed. To explore the cellular distribution of STAT1 in human tissues and organs, we analyzed single‐cell sequencing of the human protein atlas database and found STAT1 was mainly distributed in macrophages and glandular and luminal cells in the endometrium (Figure S5, Supporting Information). To further verify the distribution of STAT1 in different macrophage subtypes, we collected IUA tissues on 3rd day after surgery and conducted a macrophage subtype‐pSTAT1 localization experiment. The results demonstrated that pSTAT1 was mainly expressed in M1 macrophages rather than M2 macrophages in IUA tissues (Figure 1F). M1 macrophage often secreted IL‐1β and TNF‐α to participate in excessive inflammation. Therefore, the transcription factor binding sites of STAT1 in IL‐1β and TNF‐α were predicted by Eukaryotic Promoter Database (EPD) and found that the sites of STAT1 were801,596 and509 in IL‐1β and777,512 and360 in TNF‐α (Figure 1G). Furthermore, the concentration of IL‐1β and TNF‐α were detected during the IUA course in the rat model (0, 1, 3, 7, 14, 28 days after injury). As shown in Figure 1H, in vivo experiments indicated a synergistic effect between IL‐1β and TNF‐α, reaching peak expression at 3 days postoperatively. Therefore, in this study, we chose the 3rd and 28th day postoperatively as the observation point to assess the anti‐inflammation and anti‐adhesion effect of STAT1 inhibition in M1 macrophages.

### 2.2 Construction and Validation of FA‐EVs

**Figure 2:**
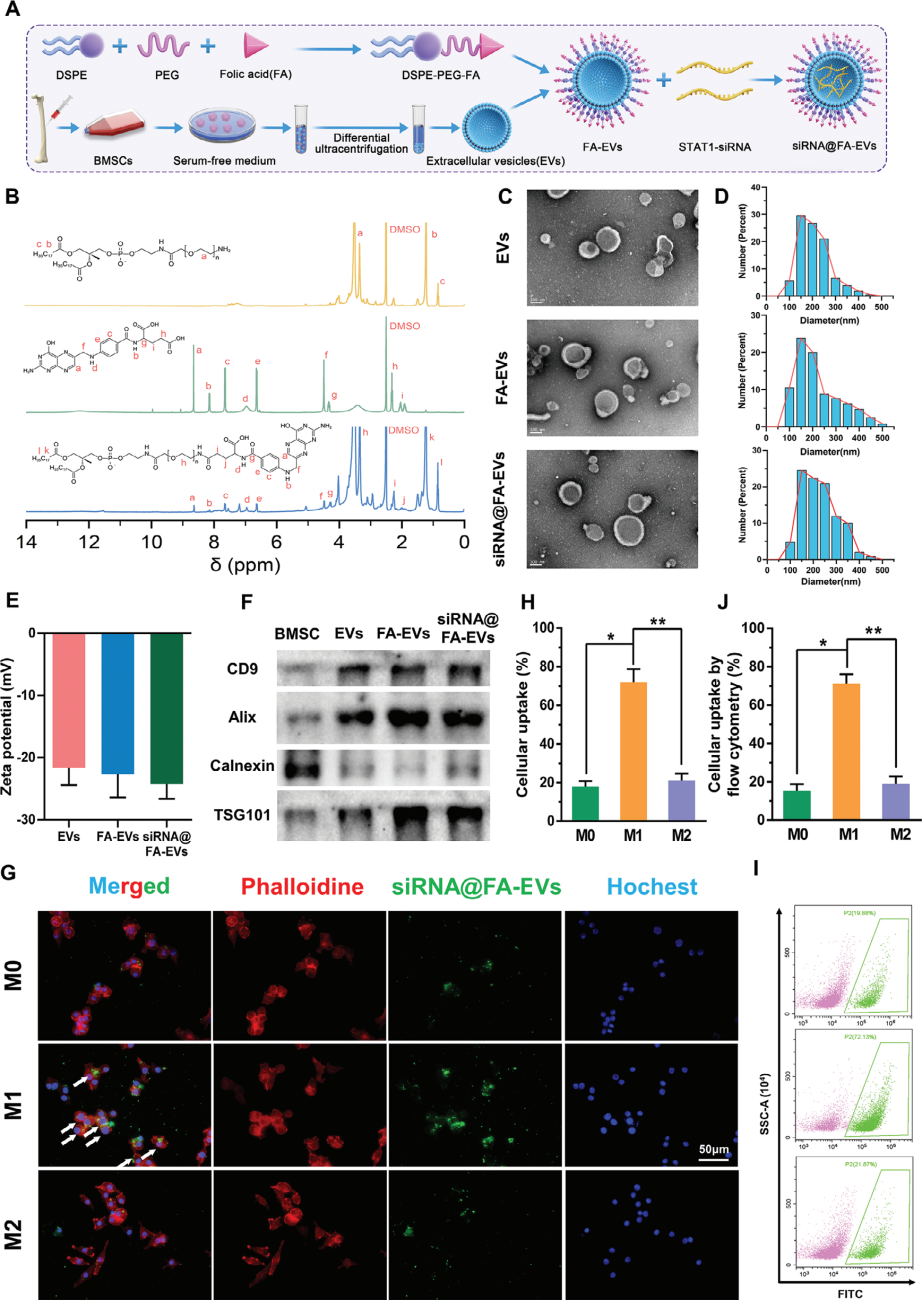
A) The synthesis process of siRNA@FA‐EVs. B) 1H‐NMR of DSPE‐PEGFA. C) The morphology of EVs and FA‐EVs were detected by TEM. D, E) Particle size and zeta potential of EVs, FA‐EVs and siRNA@FA‐EVs. F) Detection of extracellular vesicle biomarkers of BMSC, EVs, FA‐EVs, and siRNA@FA‐EVs. G,H) Fluorescence images and cellular uptake of different subtypes (M0, M1, and M2) macrophages treated with FA‐EVs. I,J) Flowcytometric assays of siRNA@FA‐EVs uptake by different subtypes of macrophages. Data are presented as mean ± SD. *p < 0.05 compared to the M0 group, **p < 0.05 compared to the M2 group, *n =* 6.

BMSCs were isolated from bone marrow and we applied ultra‐high‐speed centrifugation to extract EVs from BMSCs. Folate receptor (FR) was previously reported that mainly distributed on the membrane surface of M1 polarized macrophages.^[^ ^31^ ^]^ FA could bind specifically to FR on the surface of M1 macrophages, and then help the modified vehicle to be precisely swallowed and internalized for M1 macrophage‐targeted delivery.^[^ ^32^ ^]^ In order to efficiently regulate macrophage M1 polarization, FA‐modified EVs (FA‐EVs) were constructed as described in the Experimental Section. As illustrated in **Figure** **2**A, pyridine and EDCI were added to the FA/DMSO solution, followed by DSPE‐PEG‐NH_2_. Finally, DSPE‐PEG‐FA was obtained by vacuum freeze‐drying (Figure S6, Supporting Information).

The EVs obtained were dispersed in PBS and the DSPE‐PEG‐FA solution was added and fully reacted to obtain FA‐EVs. DSPE‐PEG‐FA has excellent affinity with phospholipid bilayers of EVs and was used to modify EVs with FA by self‐assembly. The self‐assembly approach relies on noncovalent forces, making it a bio‐friendly strategy that does not disturb the biological activity of the engineered EVs. Purified DSPE‐PEG‐FA, DSPE‐PEG‐NH_2,_ and FA products were characterized through ^1^H nuclear magnetic resonance (^1^H NMR) spectra (Figure 2B). For DSPE‐PEG‐FA, the characteristic peaks at 0.8–3.5 ppm were assigned to the guanidine group of FA, indicating that FA was successfully conjugated to DSPE‐PEG‐NH_2_. Afterward, the morphology of EVs, FA‐EVs, and siRNA@FA‐EVs were evaluated by transmission electron microscope (TEM) images (Figure 2C) and appeared as typical bilayer membrane vesicles. Furthermore, the particle size and zeta potential of EVs, FA‐EVs, and siRNA@FA‐EVs were analyzed by dynamic light scattering with Malvern Zetasizer Nano ZS90. The particle size histogram exhibited that the average particle sizes of EVs, FA‐EVs, and siRNA@FA‐EVs were 208.4, 220.3, and 227.1 nm (Figure 2D). The Zeta potential of EVs, FA‐EVs, and siRNA@FA‐EVs were 21.60 ± 2.50,22.7 ± 3.40, and 24.27 ± 2.36 mV respectively, and displayed negative surface charge (Figure 2E). Moreover, western blotting results exhibited the EVs markers (CD9, Alix, and TSG101) were abundant in EVs, FA‐EVs, and siRNA@FA‐EVs, and non‐EVs markers (Calnexin) were not present (Figure 2F). The above results demonstrated that the successful extraction of BMSCs‐derived EVs, and more importantly, the insertion of DSPE‐PEG‐FA and the electroporation of siRNA does not affect EVs morphology and biological properties. Gene therapy is considered a potential therapeutic approach, which can inhibit the expression of specific genes to intervene in disease development and progression due to its high specificity.^[^ ^33^ ^]^ Transfection siRNA into macrophages to block their excessive M1 polarization is considered to be an effective way to successfully inhibit adhesion formation.^[^ ^34^ ^]^ However, the unstable structure of siRNA is easily degraded by RNase in vivo, resulting in poor therapeutic effect.^[^ ^35^ ^]^ In addition, siRNA is not inherently cell‐targeted, leading to a range of side effects.^[^ ^36^ ^]^ Naturally, RNA carriers, such as EVs, are to be exploited for therapeutic applications and should be modified to achieve intracellular delivery to specific tissues and organs.^[^ ^37^ ^]^ In order to precisely inhibit STAT1 expression in M1 macrophages, we first screened the STAT1‐siRNA with the highest interference efficiency in vitro (Figure S7, Supporting Information) and FAM‐labeled STAT1‐siRNA loaded FA‐EVs (siRNA@FA‐EVs) were prepared by electroporation technology. The concentration of STAT1‐siRNA in siRNA@FA‐EVs at 45.2 ng µL. To confirm the macrophage subtype targeting of siRNA@FA‐EVs, we investigated the cellular uptake of siRNA@FA‐EVs by different subtypes of macrophages (M0, M1, and M2 subtypes). The results exhibited that the intensity of green fluorescence within the M1 macrophage group was significantly higher than that of the M0 macrophage and M2 macrophage groups (Figure 2G,H), indicating that siRNA@FA‐EVs could bind precisely to FR on the surface and was more likely to be endocytosed by M1 macrophages. Additionally, flow cytometry results also indicated the uptake rate of siRNA@FA‐EVs reached 70% in M1 macrophages was significantly higher than ≈20% in M0 and M2 macrophages (Figure 2I,J). In addition, the cellular uptake rate of M1 macrophage in siRNA@FA‐EVs group was superior to that of commercialized Lipofectamine 2000 (Lipo 2000) and unmodified EVs (Figure S8, Supporting Information). Meanwhile, we also detected the uptake rate of siRNA@FA‐EVs by endometrial epithelium cells, endometrial stromal cells, and vascular endothelial cells. The results showed that the intensity of green fluorescence was weaker within each of these cell groups (Figure S9, Supporting Information). Therefore, the above results collectively suggested that siRNA@FA‐EVs have various properties including M1 macrophage targeting, high transfection efficiency, and good biocompatibility to serve as a promising vehicle for siRNA delivery and Gene therapy of M1 macrophages.

Currently, researchers applied surface modification methods to construct engineered EVs for the treatment of various diseases.^[^ ^38^ ^]^ However, the application of engineered EVs for the treatment of IUA has been rarely studied. Liu et al. used mesenchymal stem cell‐derived exosome to repair uterine injury.^[^ ^39^ ^]^ Zhao reported that exosome derived from adipose mesenchymal stem cells could restore functional endometrium in a rat model of IUA.^[^ ^40^ ^]^ In this study, targeted recognition delivery and accurate intervention of M1 macrophages was achieved with FA modification of EVs surface and loaded with exogenous STAT1‐siRNA by electroporation. This all‐in‐one EVs‐based FA‐modified targeted delivery system may be a promising therapeutic approach for IUA treatment.

### 2.3 Construction and Detection of siRNA@FA‐EVs/GS Hydrogel

**Figure 3:**
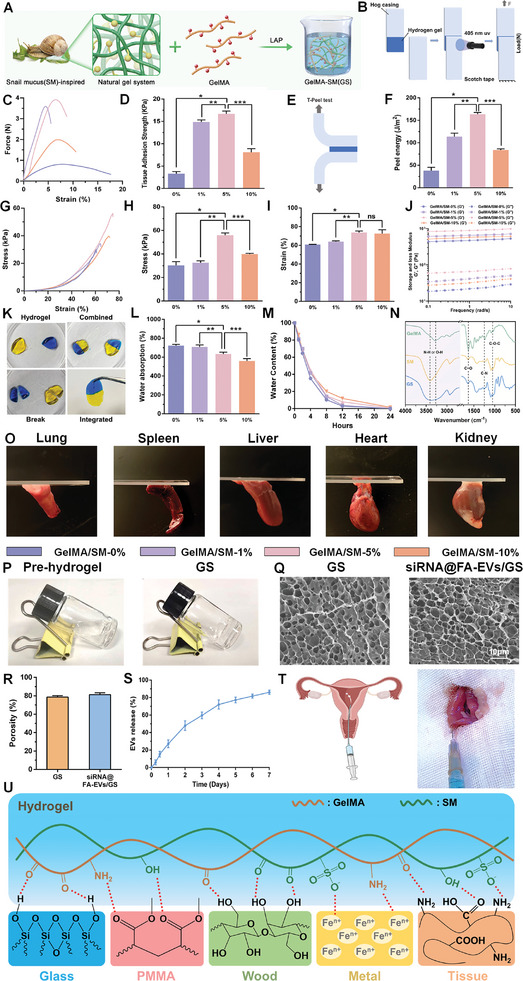
A) The synthesis process of GS hydrogel. B) The scheme of the lap‐shear experiment. C,D) Force‐strain curve and adhesive strength of hydrogels. E,F) Graphics indicated the 180° peeling test experiment and the peel energy of GelMA hydrogel with different contents of SM component; G) Compression properties of hydrogels; H, I) The maximum stress and strain of GelMA/SM (0, 1, 5, 10%). J) The rheological properties of GelMA/SM (0, 1, 5, 10%). G’: Storage modulus, G”: loss modulus. K) Integration of two broken hydrogel pieces after mutual interaction. L, M) Water absorption and retention of these hydrogels. N) FTIR spectrum of GS hydrogel. O) Visual experiment for the bio‐adhesion property of GS hydrogel. P, Q) Hydrogel formation and SEM images of GS and siRNA@FA‐EVs/GS hydrogel. R, S, T) The porosity, EVs release, and injectability of siRNA@FA‐EVs/GS hydrogel into the uterine cavity. U) The sustained release curve of siRNA@FA‐EVs from hydrogel. N) Probable adhesive mechanism of the hydrogel to different substrates. Data are presented as mean ± SD. *p < 0.05 compared to the GelMA/SM‐0% group, **p < 0.05 compared to the GelMA/SM‐1% group, ***p < 0.05 compared to the GelMA/SM‐10% group, *n =* 3.

Currently, anti‐adhesion hydrogels commonly used in the clinical treatment of IUA are prone to slip off from injury sites due to poor bio‐adhesion properties, and they have insufficient interaction time to achieve long‐lasting physical barrier effects to stop the invasion of adhesion tissue into the injury sites and the unique shape of the uterus prevents the liquid mixture remaining in the uterine cavity.^[^ ^1b^ ^]^ Above reasons result in the reduction of anti‐adhesion efficacy. Solving this challenge requires hydrogel with strong bio‐adhesion properties. In this work, bioinspired by the natural snail mucus (SM), we fabricated a novel adhesive hydrogel by combining the snail mucus with GelMA hydrogel. To screen the optimal adhesive hydrogel, GelMA (60% substitution) and different proportions of SM at 0%, 1%, 5%, and 10% (w/w) in the presence of a photopolymerization initiator were mixed for comparison (**Figure** **3**A). We expected that hydrogel could attach to wet tissue with high adhesion, due to 3D double network structure through abundant hydrogen and ionic bonds. To verify our hypothesis, tissue adhesion of the gradient component hydrogels was detected. As shown in Figure 3B,C, the adhesive strength of GelMA/SM‐1% (14.86 ± 0.45 kPa), GelMA/SM‐5% (16.69 ± 0.62 kPa) and GelMA/SM‐10% (8.10 ± 0.80 kPa) on the porcine skins was significantly higher than that of GelMA‐SM‐0% (3.31 ± 0.46 kPa) in the lap‐shear experiment, suggesting that SM incorporation could effectively enhance bio‐adhesion property of hydrogels. At 5% SM concentration, the hydrogel exhibited prominently stronger adhesion than others (Figure 3D). To measure the work of adhesion performance in the normal direction, we performed 180°peeling adhesion resistance tests (Figure 3E). Among them, the peel strength of GelMA/SM‐5% hydrogel reached up to 163.4 J m^−2^, which was consistent with the result of the lap shear test. In subsequent experiments, pig uterus instead of skin should be used to better reflect the bio‐adhesion of the hydrogel to the uterus. Meanwhile, the bio‐adhesion property of GS hydrogel was also evaluated by a visual adhesion experiment and authenticated to have great bio‐adhesion properties to tissues or organs (heart, liver, spleen, lung, and kidney). (Figure 3O). Compared with hydrogels without SM, there was tight adhesion between the siRNA@FA‐EVs/GS hydrogel and the endometrium, which indicated that siRNA@FA‐EVs/GS hydrogel has great bio‐adhesion properties to uterus in vitro and vivo (Figures S10 and S11, Supporting Information). Rheology and compression experiments indicated that SM incorporation could enhance the mechanical properties of GelMA hydrogel, especially for GelMA/SM‐5% hydrogel. The two cut pieces of hydrogel gradually form an integral hydrogel through cut surfaces recontacting without external influence, demonstrating its self‐healing property (Figure 3K). In addition, we also examined the water absorption and retention rates of these hydrogels and found that with the incorporation of SM, the water absorption of the hydrogel gradually decreased but the water retention increased (Figure 3L,M). Among them, the water absorption and retention of GelMA/SM‐5% hydrogel was moderate. The incorporation of SM provides this hydrogel with bio‐adhesion and self‐healing properties, allowing for long‐lasting coverage of the injury sites of the uterus and maintaining its structural integrity when is squeezed in the dynamic environment of the uterus (Figure S10, Supporting Information). After comprehensively considering the above results, we selected GelMA/SM‐5% and named GS for further in vitro and vivo experiments.

According to the Fourier transform infrared (FTIR) spectrum (Figure 3N), the characteristic peaks of C─O─C, C─N, and C═O stretching vibrations attributed to amide bonding appeared at 1053, 1240, and 1628 cm^−1^ for both GelMA, SM, and GS hydrogel. It is shown that GS hydrogels are rich in hydroxyl and amine groups as well as hydrogen bonding formed by carbonyl groups, which are the main drivers of the adhesion properties of the gels. Furthermore, sharp characteristic absorption peaks at 3281 and 3388 cm^−1^ were observed for GelMA and SM, corresponding to O─H and N─H stretching vibrations, respectively. In contrast, the GS hydrogel showed broad absorption peaks, indicating that GelMA and SM formed a homogeneous GS hybridization‐enhanced hydrogel. Subsequently, we added siRNA@FA‐EVs into pre‐hydrogel before light‐curing and synthesized siRNA@FA‐EVs/GS hydrogel under UV irradiation. As shown in Figure 3P,Q, the morphology and structure of GS and siRNA@FA‐EVs/GS hydrogels were evaluated by scanning electron microscope (SEM). SEM images exhibited a porous network crosslinked structure. It was found that there was no significant difference in porosity between both hydrogels (Figure 3R). Then, we established the sustained release profile of Dio‐labeled siRNA@FA‐EVs from the hydrogel and the release curve presented the burst release of siRNA@FA‐EVs in the first 3 days followed by sustained release for the subsequent 7 days, with a cumulative release amount of 86.07 ± 2.36% (Figure 3S). The sustained release of FAM‐labeled siRNA from siRNA@FA‐EVs/GS hydrogel was also measured (Figure S12, Supporting Information), which was consistent with the trend of Dio‐labeled siRNA@FA‐EVs from hydrogel. As indicated in Figure 2F, we could find that EVs markers (CD9, Alix, and TSG101) were abundant in siRNA@FA‐EVs released from siRNA@FA‐EVs/GS hydrogel and non‐EVs markers (Calnexin) were not present. In addition, siRNA@FA‐EVs/GS hydrogel in PBS solution could maintain a certain mass in 3 days, and <10% of the mass remained under‐graded after 7 days (Figure S13, Supporting Information). As demonstrated in Figure 3T, the macroscopic injectability into the uterine cavity of the siRNA@FA‐EVs/GS hydrogel was confirmed. Afterward, the Dead/Live and CCK‐8 assay further demonstrated the siRNA@FA‐EVs/GS hydrogel had good biocompatibility on macrophage under an LPS‐induced inflammatory microenvironment with no cytotoxicity (Figures S14 and S15, Supporting Information). The interaction mechanism of siRNA@FA‐EVs/GS hydrogel on the different adhesion surfaces was exhibited in Figure 3U.

Although Deng et.al had reported the application of natural bioadhesives derived from snail mucus for wound repair and anti‐inflammatory purposes, it lacked cellular targeting and could not achieve intervention of specific cell populations or genes.^[^ ^41^ ^]^ In this study, inspired by snail mucus, we prepared siRNA@FA‐EVs/GS hydrogels by mixing siRNA@FA‐EVs and GS under UV irradiation. The incorporation of siRNA@FA‐EVs was capable of targeting M1 macrophages in IUA and inhibited STAT1 phosphorylation through gene therapy, thereby promoting the resolution of inflammation and relieving IUA.

### 2.4 siRNA@FA‐EVs/GS regulates LPS‐induced Macrophage M1 Polarization and Pro‐inflammatory Release

**Figure 4:**
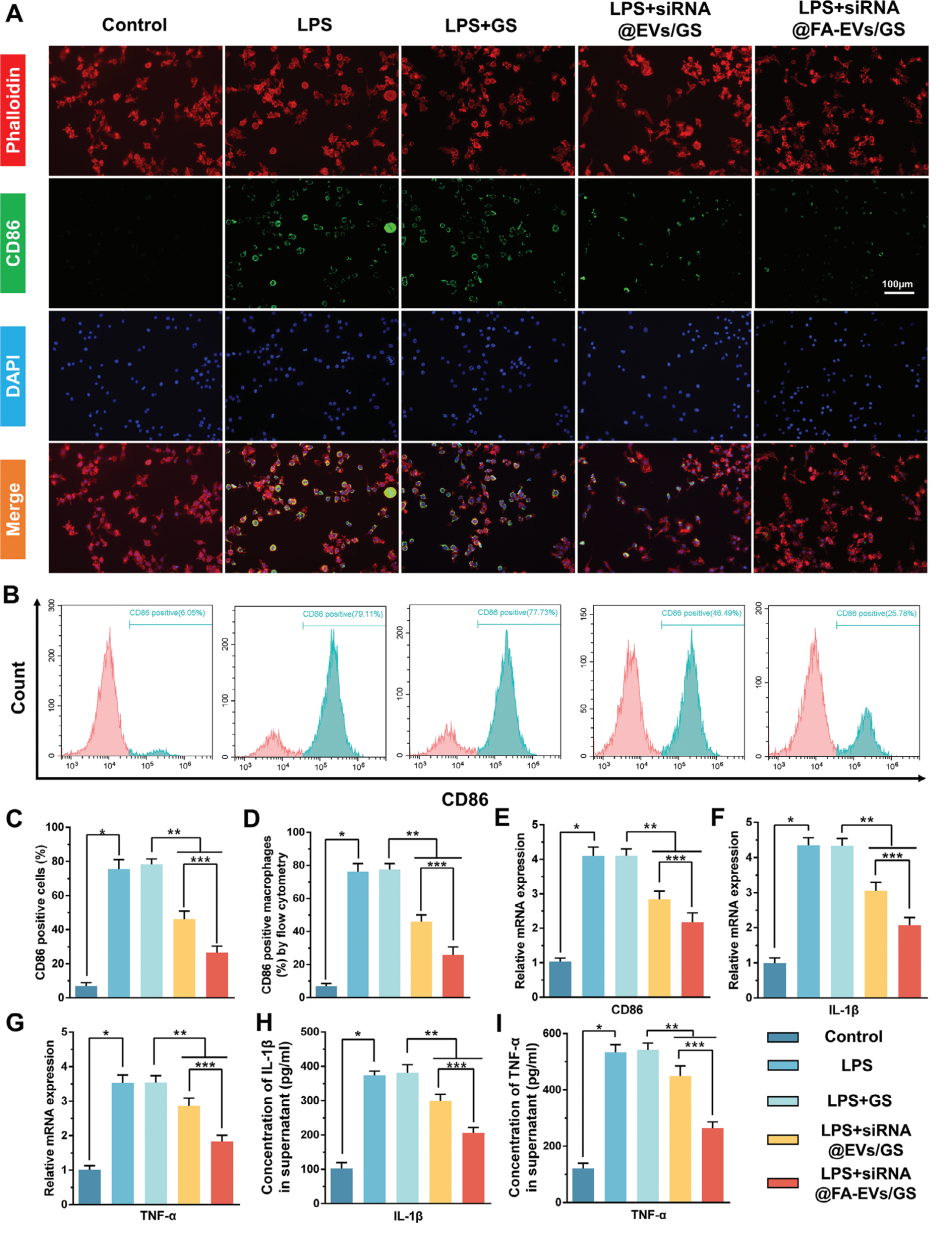
A,B) Immunofluorescence staining and flow cytometric assays for M1 macrophage polarization in diverse microenvironments. C, D) The percentage of CD86‐positive cells was calculated through immunofluorescence and flow cytometry. E, F, G) The mRNA expression of CD86, IL‐1β, and TNF‐α was detected by RT‐PCR in different groups. H, I) The concentrations of IL‐1β and TNF‐α in cell supernatant were measured by ELISA. Data are presented as mean ± SD. *p < 0.05 compared to the control group, **p < 0.05 compared to the LPS+GS group, ***p < 0.05 compared to the LPS+siRNA@EVs/GS group, *n =* 6.

The excessive adhesion tissue formation can be triggered by an inflammatory response in damaged endometrium, which is critical in the disorder of endometrial repair and the development of IUA.^[^ ^42^ ^]^ Persistent inflammatory activation coexisted with adhesion in the endometria of IUA patients and the degree of inflammation was positively correlated with that of IUA. M1 macrophage persistence along with pro‐inflammatory cytokines secretion was responsible for excessive inflammation.^[^ ^43^ ^]^ To investigate the inhibition of M1 polarization and pro‐inflammatory cytokines release by siRNA@FA‐EVs/GS, we placed hydrogels in the upper chamber of the transwell and then seeded RAW264.7 in the lower chamber, adding LPS to simulate the inflammatory microenvironment after intrauterine injury and accelerate macrophage M1 polarization. The M1 polarization of macrophages and pro‐inflammatory cytokines secretion were evaluated by mRNA and protein level detection including immunofluorescent staining, RT‐PCR, flow cytometry, and ELISA. As shown in **Figure** **4**A, the cell nucleus and cytoskeleton were stained with Hoechst (blue) and phalloidine (red), and CD86 fluorescence was displayed in green. The level of macrophage M1 polarization was assessed by counting the percentage of CD86‐positive (a special biomarker of M1 subtype) macrophages. The results indicated that siRNA@EVs/GS could significantly relieve LPS‐induced macrophage M1 polarization as represented by a decreased percentage of CD86‐positive macrophage in the injury‐state microenvironments compared with the LPS+GS group (Figure 4C). The SM in GS gel could not affect macrophage M1 polarization due to its low content (Figure S16, Supporting Information). More importantly, the percentage of CD86‐positive macrophage in the LPS+siRNA@FA‐EVs/GS group was less than that in the LPS+siRNA@EVs/GS group, which suggested that FA‐EVs could achieve accurate identification and targeted intervention on macrophage M1 polarization. In addition, flow cytometry results provided more accurate quantification and also supported this finding (Figure 4B,D). The RT‐PCR and ELISA were applied to detect the transcriptional and protein level of downstream effects after macrophage M1 polarization. The CD86 and pro‐inflammatory cytokines related to M1 polarization involved with IL‐1β and TNF‐α were measured. In the LPS and LPS+GS groups, the levels of CD86, IL‐1β, and TNF‐α significantly increased and no significant difference was observed between the two groups. However, LPS‐induced macrophage treated with siRNA@EVs/GS exhibited an obviously decreased mRNA and protein level of CD86, IL‐1β, and TNF‐α. Moreover, we also detected the effect of unmodified EVs‐loaded GS hydrogel on inflammatory cytokines release and found that siRNA@FA‐EVs/GS amplified the inhibitive effect on inflammatory cytokines secretion through precise targeting and gene intervention (Figure S17, Supporting Information). The above results of the in vitro experiment provided powerful support for the hypothesis that siRNA@FA‐EVs/GS could exert a stronger anti‐inflammatory property compared with siRNA@EVs/GS, thereby offering endometrium local microenvironment conducive to regeneration and inhibiting macrophage M1 polarization.

### 2.5 siRNA@FA‐EVs/GS inhibits STAT1 Phosphorylation in M1 Macrophages

**Figure 5:**
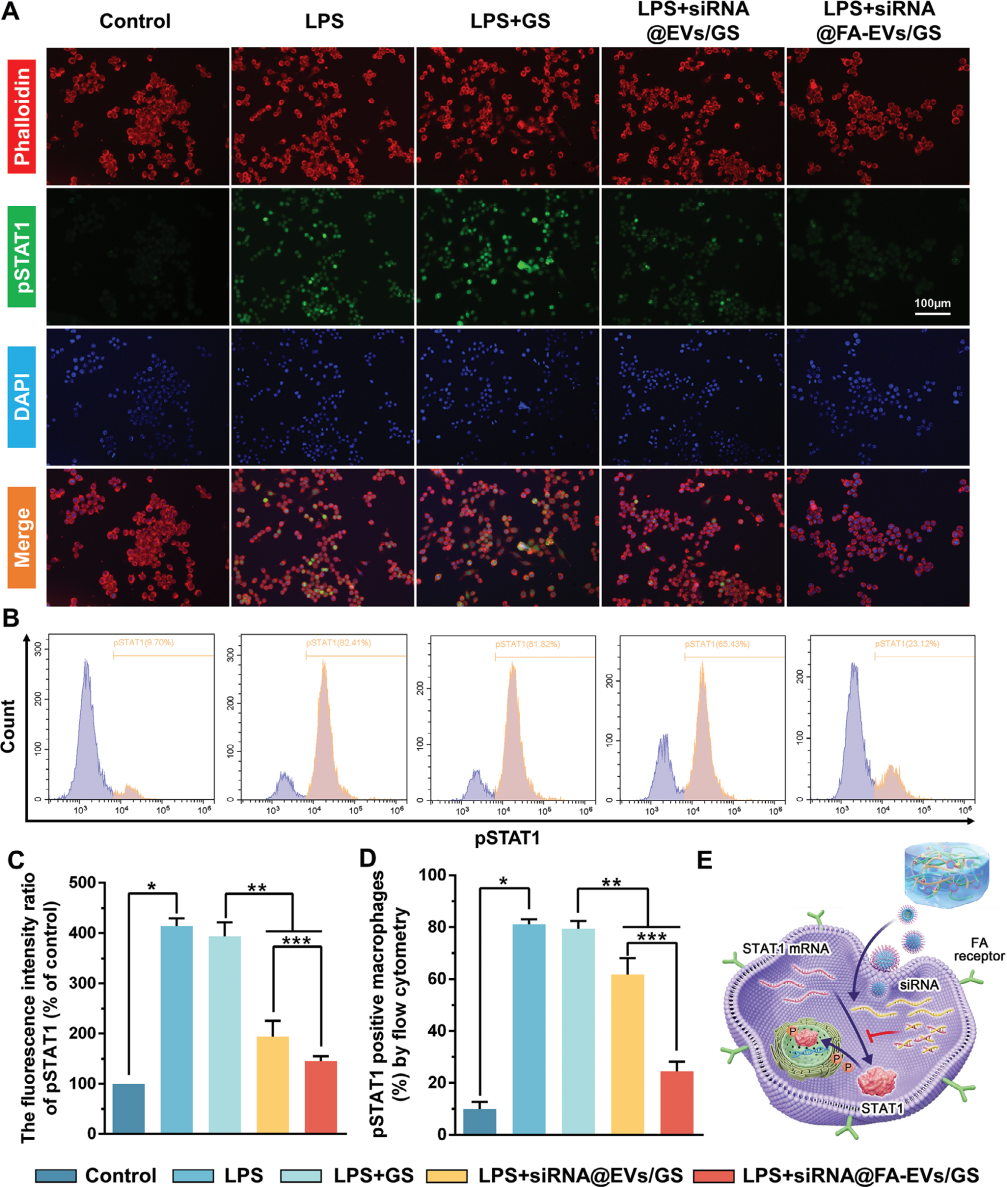
A,B) The phosphorylation level of STAT1 assessed by fluorescence microscopy and flow cytometric assays. C) The fluorescence intensity ratio of pSTAT1 in the nucleus of macrophages. D) The percentage of pSTAT1‐positive macrophages was detected by flow cytometry. E) Schematic representation of siRNA@FA‐EVs/GS inhibited M1 macrophage polarization by inhibiting STAT1 phosphorylation. Data are presented as mean ± SD. *p < 0.05 compared to the control group, **p < 0.05 compared to the LPS+GS group, ***p < 0.05 compared to the LPS+siRNA@EVs/GS group, *n =* 6.

Macrophage M1 polarization has been demonstrated to be closely linked to the STAT1 signaling pathway. Phosphorylated STAT1 into the nucleus, which in turn leads to macrophage M1 polarization and the release of pro‐inflammatory and pro‐fibrotic cytokines, has been reported in a variety of fibrosis diseases but not in IUA.^[^ ^44^ ^]^ Therefore, we speculated that the STAT1 may participate in IUA by mediating M1 macrophage polarization and pro‐inflammatory cytokines secretion. The phosphorylated levels of STAT1 were evaluated by immunofluorescence and flow cytometry. As indicated in **Figure** **5**A, immunofluorescence staining exhibited the phosphorylation levels of STAT1 in differently treated macrophages, and the average fluorescence intensity in the nucleus was measured. The results demonstrated that the green fluorescence (pSTAT1) was the weakest observed inside the cell's nucleus of the control group. There was no significant difference in the intensity of green fluorescence observed inside the cell nucleus in the LPS and LPS+GS groups. Among the LPS+GS group, LPS+siRNA@EVs/GS group, and LPS+siRNA@FA‐EVs/GS group, the green fluorescence observed inside the cell nucleus of the LPS+siRNA@FA‐EVs/GS group was the weakest (Figure 5C). These observations supported our hypothesis that siRNA@FA‐EVs/GS effectively inhibited STAT1 phosphorylation. The expression of pSTAT1 was detected by flow cytometry, and the results were consistent with the above (Figure 5B,D). The above findings supported our hypothesis that siRNA@FA‐EVs/GS inhibited LPS‐induced M1 polarization and pro‐inflammatory factor release from macrophages by suppressing STAT1 phosphorylation, thus reducing the inflammatory response toward intrauterine injury and blocking the formation of IUA (Figure 5E).

Previous research related to IUA paid excessive attention to the promotion of the M1 to M2 subtype switch to achieve the shift from an inflammatory toward an anti‐inflammatory microenvironment.^[^ ^45^ ^]^ However, these studies have ignored the underlying mechanism of dysfunctional inflammatory response caused by intrauterine injury. Therefore, revealing the pivotal target‐mediated IUA during the inflammatory stage could effectively relieve excessive inflammation induced by macrophage M polarization. In this study, we first elucidated excessive STAT1 phosphorylation facilitated intrauterine inflammation and aggravated IUA formation. siRNA@FA‐EVs/GS hydrogel in this study could obviously inhibit macrophage M1 polarization pro‐inflammatory cytokines release by intercepting transcriptional activation promotion of STAT1, which concluded that siRNA@FA‐EVs/GS hydrogel had high hopes for being a potential alternative for IUA clinical treatment.

### 2.6 siRNA@FA‐EVs/GS Inhibited the Transformation of ESCs into Myofibroblasts Mediated by Macrophage M1 Polarization

**Figure 6:**
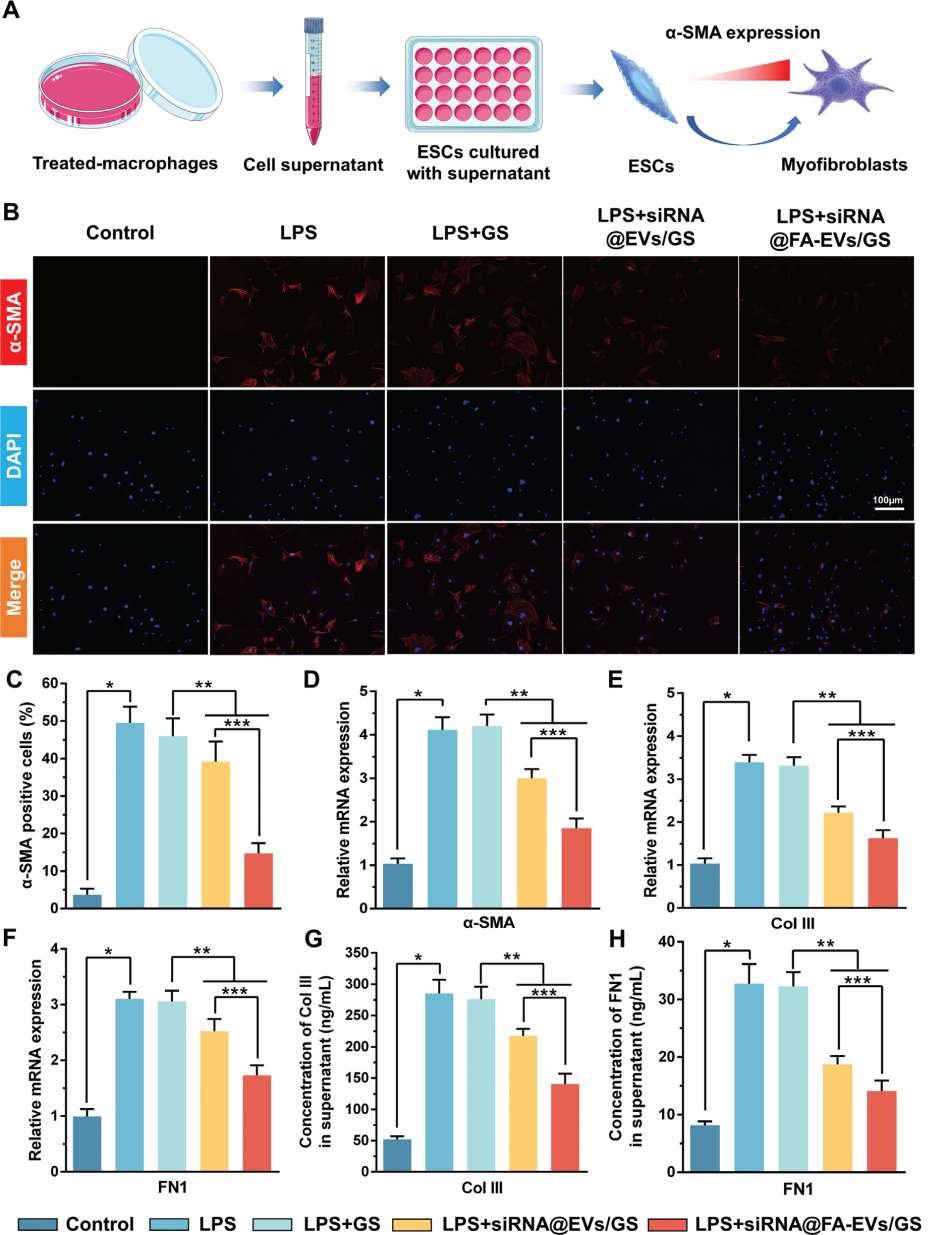
A) The schematic diagram of crosstalk between treated macrophages and ESCs. B,C) The percentage of α‐SMA positive cells evaluated by immunofluorescence staining in each group. D–F) The mRNA expression of α‐SMA, Col III, and FN1 was detected by RT‐PCR in different groups. G,H) The concentrations of Col III and FN1 in cell supernatant were measured by ELISA. Data are presented as mean ± SD. *p < 0.05 compared to the control group, **p < 0.05 compared to the LPS+GS group, ***p < 0.05 compared to the LPS+siRNA@EVs/GS group, *n =* 6.

The occurrence and development of IUA is a complex pathological process. We have reported that the formation of adhesion tissues in IUA is mostly related to the excessive myofibroblast activation and deposition within the uterine cavity.^[^ ^46^ ^]^ As the injury‐triggered inflammation subsides, the transformation between ESCs into myofibroblasts plays a prominent role in the progress of IUA.^[^ ^47^ ^]^ To further investigate the intercellular regulatory mechanisms between macrophage M1 polarization and ESCs transformation, ESCs were cultured with treated‐macrophage supernatant and detected the cell transformation and extracellular matrix deposition including Col III and FN1 (**Figure** **6**A). After 24 h culture, immunofluorescence staining results demonstrated that the percentage of α‐SMA‐positive cells and mRNA expression were enhanced in LPS and LPS+GS groups as compared with the control groups (Figure 4B–D). In contrast, the LPS+siRNA@EVs/GS group and LPS+siRNA@FA‐EVs/GS group exhibited less ESCs transformation and lower expression of α‐SMA. Especially, siRNA@FA‐EVs/GS could indeed inhibit myofibroblast activation with only a 13.74±3.04% positive rate to the greatest extent. Furthermore, siRNA@FA‐EVs/GS also suppressed inflammation‐induced mRNA expression and secretion of Col III and FN1 from myofibroblasts (Figure 6E–H).

Precise therapy of inflammatory cascade mediated by macrophage polarization in IUA has become the research hotspot. However, IUA caused by chronic inflammation directly strangles the reproductive health of women. Liu et al fabricated a hydrogel membrane with a negative surface potential to absorb pro‐inflammatory cytokines with positive potential.^[^ ^48^ ^]^ However, the underlying interaction between inflammatory microenvironment and adhesion formation was not elucidated. In our study, we can preliminarily conclude that STAT1 phosphorylation‐mediated macrophage M1 polarization and pro‐inflammatory factor release induces the transformation of ESCs to myofibroblasts and the release of collagen III and FN1, which accelerates the formation of adhesion tissues. siRNA@FA‐EVs/GS effectively reversed these results by gene therapy inhibiting STAT1 phosphorylation in M1 macrophages.

### 2.7 siRNA@FA‐EVs/GS Hydrogel Inhibited Intrauterine Adhesion

Although anti‐adhesion HA hydrogel is one of the commonly used clinical treatments for IUA, it only serves as a physical barrier and lacks biological activity and bio‐adhesion properties, resulting in poor anti‐adhesion effects. In order to investigate the anti‐adhesion property of siRNA@FA‐EVs/GS hydrogel in the IUA, we next established a model of endometrial damage by scratching the endometrium (Figure S18, Supporting Information). Intraoperatively, the rats were seen to have a “Y” shaped double uterus, which was smooth, red, and elastic before surgery, and the uterus was obviously congested and slightly hard after scratching the cavity. After scrabbing, it was easily observed that the thickness of the wounded uterus became thinner with bleeding. Subsequently, except for the IUA group, the other groups were injected with different compositions of hydrogel by syringe into the uterine cavity. After rats were sacrificed on the 28th day postoperatively, the degree of adhesion was assessed using gross observation and histological analysis. We observed that the uterus of the IUA group was seen to be distorted, with total obstruction of the uterine cavity. The uterine cavity of GS group, siRNA@EVs/GS, and siRNA@FA‐EVs/GS group exhibited partial obstruction, partial obstruction, and clear respectively. In addition, histological evaluation indicated that endometrium injury caused poor morphologic recovery as evidenced by the reduction of endometrial thickness and number of glands and the increase of adhesion area. On the contrary, GS hydrogel and siRNA‐loaded EVs could effectively promote endometrium repair and relieve adhesion formation. Especially, the siRNA@FA‐EVs/GS group presented thicker regenerated endometrium, more glands, and less adhesion than siRNA@EVs/GS, indicating that M1 macrophage‐targeted intervention with STAT1 can observably inhibit the progress of IUA formation. In addition, we also compared the therapeutic effects of clinical HA hydrogel and GS hydrogel on IUA in rats, and the results showed that the IUA+GS group had less adhesion than the IUA+HA group (Figure S19, Supporting Information). This was attributed to the stronger bio‐adhesion property of GS hydrogel that could cover the injury sites for a long time to act as the physical barrier, while HA hydrogel with poorer bio‐adhesion property was prone to slippage without sufficient time to block myofibroblast invasion.

**Figure 7:**
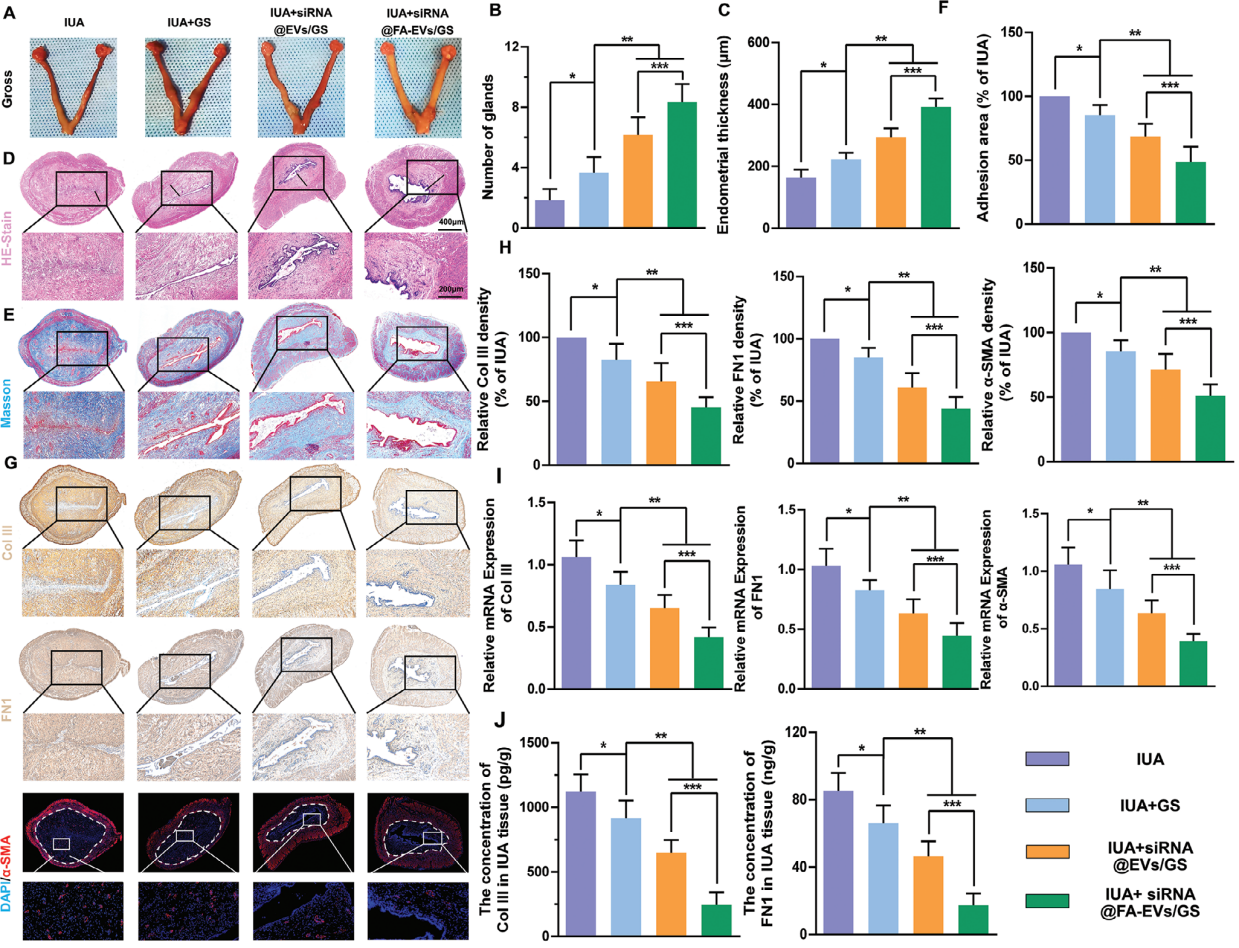
A) The gross observation of IUA sections after 28 days of surgery. B,C) Glands number and Endometrial thicknesses in each group. D) Representative images of H&E of rat uteri sections at 28 days postoperatively. E,F) Masson staining images and adhesion area were calculated. G, H) Immunohistochemical and immunofluorescence staining for Col III, FN1, and α‐SMA in IUA tissues. I) mRNA expression of Col III, FN1, and α‐SMA was recorded by RT‐PCR. J) The release of Col III and FN1 was evaluated by ELISA on the postoperative 28th day. Data are presented as mean ± SD. *p < 0.05 compared with IUA; **p < 0.05 compared with IUA+GS group. ***p < 0.05 compared to the IUA+siRNA@EVs/GS group, *n =* 6.

The essence of IUAs is fibrosis seen as excessive extracellular matrix deposition including Col III and FN1. To detect expression changes of IUA‐related proteins, immunohistochemical and immunofluorescence staining were applied to evaluate the density of Col III and FN1 and the percentage of 𝛼‐SMA (the myofibroblast biomark) positive cells. As shown in **Figure** **7**G, staining results indicated that siRNA@FA‐EVs/GS could effectively decrease the deposition of Col III and FN1 and myofibroblast transformation compared to the other three groups. Likewise, RT‐PCR and ELISA results also demonstrated that siRNA@FA‐EVs/GS had a superior therapeutic effect on IUA, suggesting its significant role in suppressing myofibroblast transformation and excessive deposition of extracellular matrix around injury sites through regulating inflammatory microenvironment.

To our current understanding, while intrauterine EVs injections in rats require abdominal incision, siRNA@FA‐EVs/GS hydrogel injection presents a promising non‐invasive alternative for treating IUA in human patients. Administering the hydrogel through the cervix using a soft tip catheter and guiding its placement into the uterus via ultrasound to minimize the risk of endometrial injury and streamline the operation. However, further large‐scale clinical trials are essential to fully evaluate the efficacy of this approach and its potential as a viable treatment option for IUA.

### 2.8 siRNA@FA‐EVs/GS Hydrogel Promotes Functional Recovery of the Uterus

**Figure 8:**
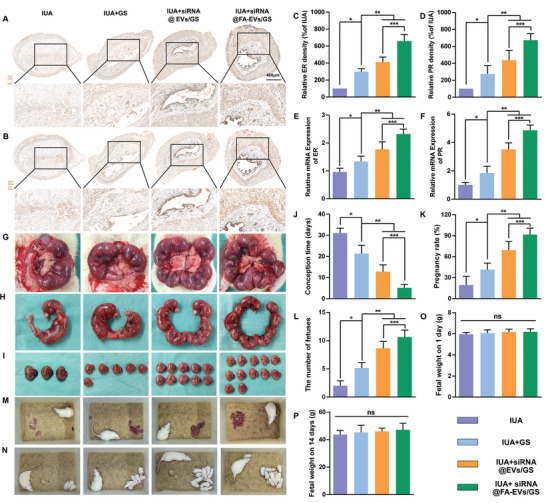
A,B) Immunohistochemical staining of ER and PR in regenerated endometrium. C,D) The semi‐quantitative analysis of ER and PR protein expression. E,F) mRNA expression of ER and PR was detected by RT‐PCR. G‐I‐) The pregnant uterus and SD rat fetuses in different groups. J,K) The conception time and pregnancy rate of SD rats with different treatments. L) The number of fetuses. M,N) Photographic images of newborn rats and after feeding 14 days. O, P) The fetal weight on the first day and 14th day. Data are presented as mean ± SD. *p < 0.05 compared with IUA; **p < 0.05 compared with IUA+GS; ***p < 0.05 compared with IUA+siRNA@EVs/GS group, ns, no significance between two groups, *n* = 6.

Estrogen and progesterone are well‐established inducers of estrogen receptor α (ER) and progesterone receptor (PR) expression in the endometrial tissue. As pivotal markers of endometrial receptivity, ER and PR are commonly used to evaluate the therapeutic effectiveness of interventions aimed at restoring uterine function. To provide supporting data on functional recovery of the uterus, we performed immunohistochemical staining and RT‐PCR to detect the protein and mRNA expression of ER and PR in the uterine after the 28th day operatively (**Figure** **8**A,B). Based on the results we found that the increased transcriptional and translational levels of ER and PR were observed in the IUA+siRNA@EVs/GS and IUA+siRNA@FA‐EVs/GS groups compared with IUA and IUA+GS group (Figure 8C–F). Especially, treatment with siRNA@FA‐EVs/GS exhibited obvious expression of both ER and PR, emphasizing the potent pro‐regenerative potential.

The main way to evaluate the restoration of endometrial function was fertility. In our study, we performed a series of fertility experiments to examine the recovery of the uterus in rats with uterine injury. The rats were mated 28 days after surgery and euthanized at mid‐to‐late gestation. Then we collected uterus for analysis of reproductive outcomes. As shown in Figure 8G–L, the pregnancy rate and the number of fetuses in rats treated with siRNA@FA‐EVs/GS were much higher than those in the other groups. The conception time in rats treated with siRNA@FA‐EVs/GS was much shorter than those in the other groups. Furthermore, we observed the growth and development of the progeny in the group of siRNA@FA‐EVs/GS over a 14‐day period and noted that they exhibited normal neonatal growth patterns (Figure 8N). However, there were no significant differences in the average weight of fetuses between different groups (Figure 8O,P). The above results showed that siRNA@FA‐EVs and GS hydrogels synergistic treatment could promote functional endometrial regeneration and restore fertility.

### 2.9 siRNA@FA‐EVs/GS Hydrogel Regulated Inflammatory Microenvironment of IUA

**Figure 9:**
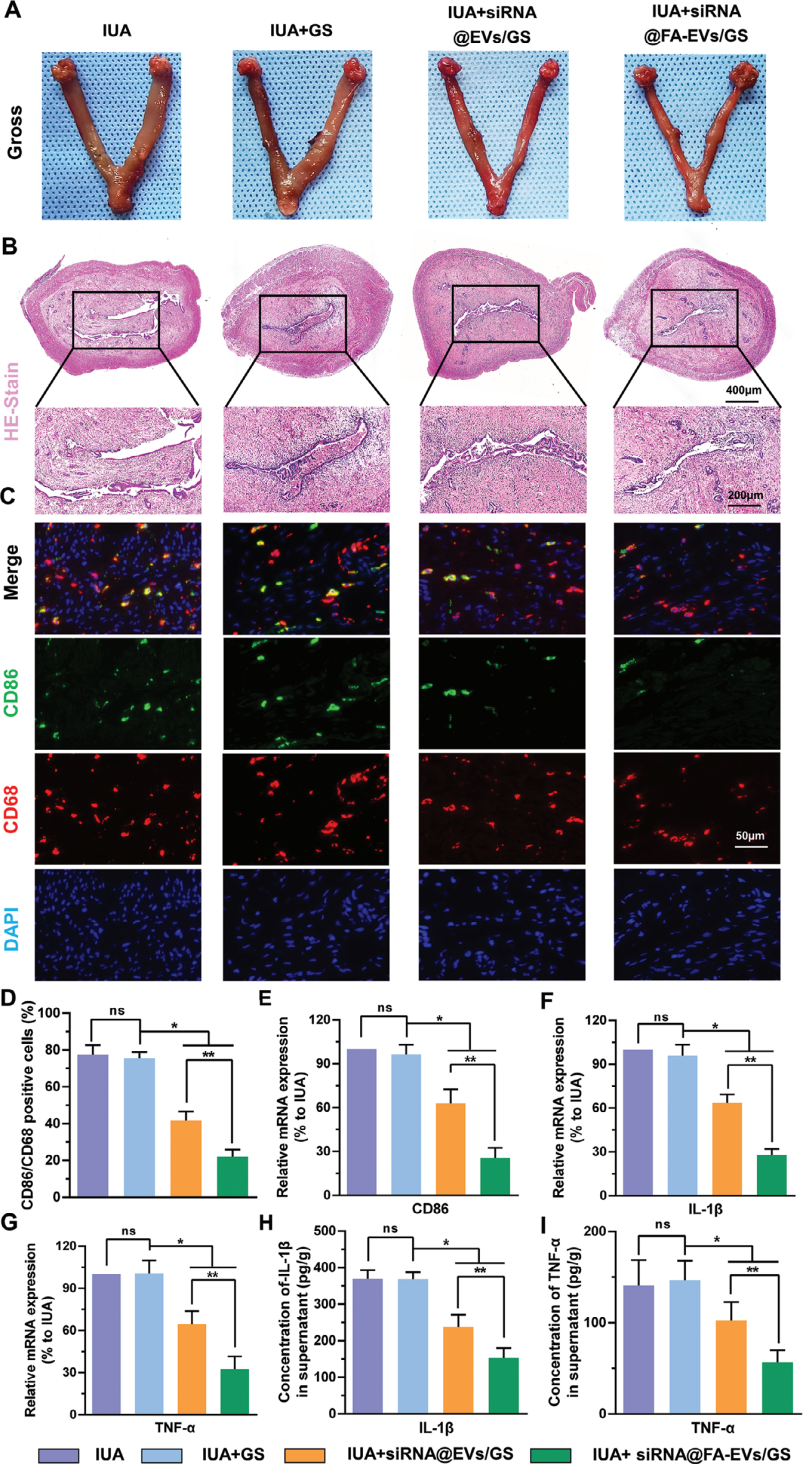
A) The gross observation of injury sites after 3 days of surgery. B) Representative images of H&E of injury sites at 3 days postoperatively. C) The immunofluorescence staining for CD86/CD68 in IUA tissues. D) The percentage of CD86/CD68 positive cells evaluated by immunofluorescence staining in each group. E–G) The mRNA expression of CD86, IL‐1β, and TNF‐α was recorded by RT‐PCR. H, I) The release of IL‐1β and TNF‐α was evaluated by ELISA on the postoperative 28th day. Data are presented as mean ± SD. *p < 0.05 compared with IUA+GS; **p < 0.05 compared with IUA+siRNA@EVs/GS group, ns, no significance between two groups, *n* = 6.

Infection and inflammatory exudates following endometrial damage are considered important risk factors for the development of IUA. Inflammation, tissue formation, and tissue reconstruction are important processes in IUA fibrosis repair. Studies have revealed that when the basal layer is severely damaged, the endometrium loses its ability to repair and regenerate and numerous inflammatory cells accumulate in the damaged endometrium, inducing an inflammatory response and secreting a wide range of inflammatory cytokines.^[^ ^49^ ^]^ In particular, they secrete IL‐1β and TNF‐α, which in turn promote the development of IUA. To investigate the effects of siRNA@FA‐EVs/GS hydrogel on the endometrial immune microenvironment, all rats were sacrificed and the injured uterus was evaluated under gross observation and histological analysis. As shown in **Figure** **9**A,B, the IUA and IUA+GS groups exhibited marked edema, inflammatory hyperemia, and structural deformation after curettage. Interestingly, treatment with siRNA@EVs/GS and siRNA@FA‐EVs/GS resulted in the relief of inflammatory symptoms. In this inflammatory response, macrophages were recruited from peripheral blood into injury sites and polarized into the M1 subtype. Subsequently, M1 macrophages were involved in defense against host infection and bacteria removal by secreting large amounts of pro‐inflammatory cytokines including IL‐1β and TNF‐α. Excessive inflammation is a key step accelerated by myofibroblast activation and collagen deposition. Therefore, we performed the double immunofluorescence (CD86 and CD68) to detect macrophage M1 polarization in the injured endometrium. A higher infiltration of macrophages with concurrent expression of CD86 and CD68 was observed in the IUA and IUA+GS group at 3 days postoperatively compared with IUA+siRNA@EVs/GS and IUA+siRNA@FA‐EVs/GS (Figure 9C,D). In rats that had been treated with siRNA@FA‐EVs/GS hydrogel, the degree of macrophage M1 polarization was significantly decreased, emphasizing the potent suppressive immunomodulatory potential. Furthermore, RT‐PCR and ELISA results also supported this finding. Specifically, the IUA group was observed to significantly upregulated expression of the CD86 (M1 marker), along with an increase in the expression and secretion of the pro‐inflammatory cytokines including IL‐1β and TNF‐α (Figure 9E–I). On the contrary, when IUA rats were injected with siRNA@FA‐EVs/GS hydrogel, there was a remarkable decrease in the mRNA and protein expression of CD86, IL‐1β, and TNF‐α compared to the other three groups. This suggested that the injury‐triggered inflammatory microenvironment, represented by the expression and secretion of TNF‐α and IL‐1β, was inhibited by the siRNA@FA‐EVs/GS hydrogel through regulation of excessive macrophage M1 polarization.

### 2.10 siRNA@FA‐EVs/GS Hydrogel Inhibited Inflammatory Cascade Through Intervention of STAT1 Phosphorylation in IUA

**Figure 10:**
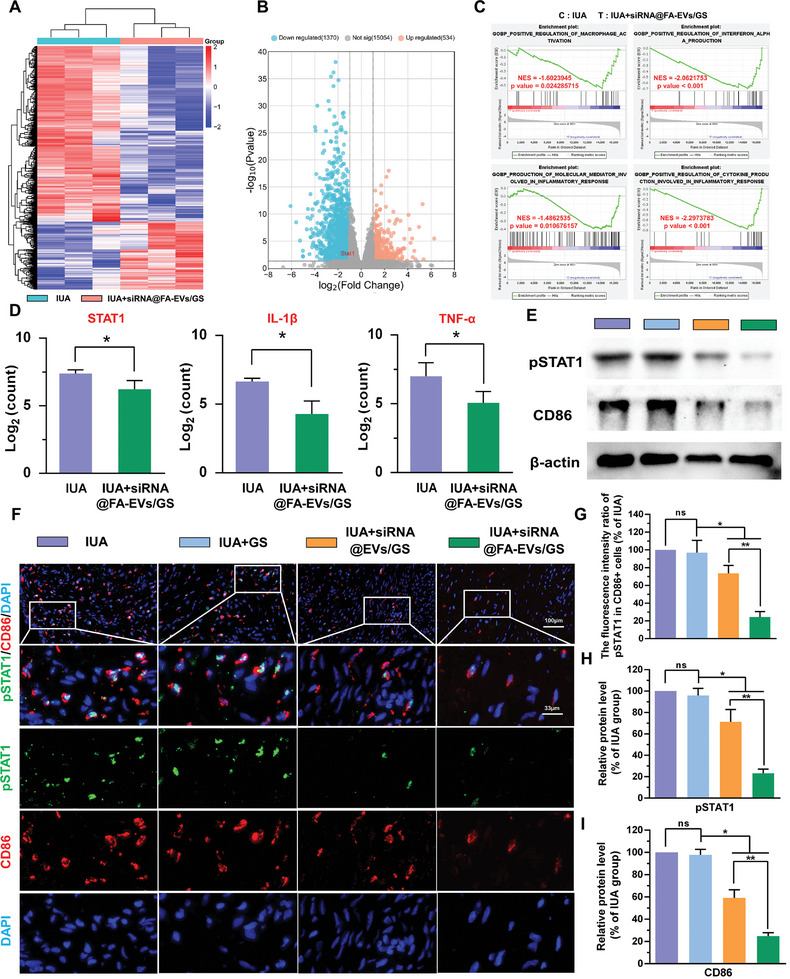
A,B) Heatmap and volcano plots of DEGs. C) GSEA analysis was applied to perform function annotation. D) The normalized count values of the STAT1, IL‐1β, and TNF‐α E) The protein bands of pSTAT1 and CD86. F) The immunofluorescence images of pSTAT1/CD86 in IUA tissues. G) The percentage of pSTAT1/CD86 positive cells evaluated by immunofluorescence staining in each group. H, I) The protein expression of pSTAT1 and CD86 was detected. Data are presented as mean ± SD. *p < 0.05 compared with IUA+GS; **p < 0.05 compared with IUA+siRNA@EVs/GS group, ns, no significance between two groups, *n* = 6.

To investigate the underlying mechanism of siRNA@FA‐EVs/GS in IUA treatment, we collected endometrial samples from IUA and IUA+siRNA@FA‐EVs/GS group after 3 days postoperatively and performed RNA‐seq analysis. Principal component analysis (PCA), gene expression density and sample‐to‐sample distance heatmaps in the quality control results indicated significant intergroup differences (Figure S20, Supporting Information). Compared with the IUA group, 537 upregulated and 1388 downregulated DEGs were identified in the IUA+siRNA@FA‐EVs/GS group through heatmap and volcano plot analysis (**Figure** **10**A,B). The protein–protein interaction (PPI) network was established based on DEGs to screen 3 hub gene modules including STAT1 (Figures S21 and S22, Supporting Information). To annotate the biological function of DEGs, GSEA analysis revealed that DEGs mainly enriched in the downregulation of positive regulation of macrophage activation, interferon alpha production, production of molecular mediator, and cytokine involved in inflammatory response (Figure 10C). Consistent with the GSEA results, the expression of pro‐inflammatory mediators including STAT1, IL‐1β, and TNF‐α was decreased in treatment with siRNA@FA‐EVs/GS (Figure 10D). Furthermore, GO enrichment analysis demonstrated that DGEs are mainly related to regulation of angiogenesis, immune receptor activity, negative regulation of myoblast differentiation, regulation of inflammatory response, response to lipopolysaccharide, leukocyte migration, and cytokine activity. KEGG results exhibited that DGEs mainly enrich in the following pathways: TNF signaling pathway, cytokine‐cytokine receptor interaction, PI3K‐Akt signaling pathway (Figures S23 and S24, Supporting Information), which are closely related to inflammation subside supported by STAT1 phosphorylation inhibition. Besides, we also performed an immune infiltration analysis and found that the proportion of M1 macrophages decreased, which suggested that STAT1 is closely associated with macrophage M1 polarization (Figure S25, Supporting Information). To explore the immune regulatory function of siRNA@FA‐EVs/GS in IUA, we performed double‐label immunofluorescence staining and western blot to assess the relationship between STAT1 phosphorylation and macrophage M1 polarization in IUA. The results exhibited the downregulated expression of pSTAT1 (green area) and CD86 (red area) distributed in injury sites in siRNA@EVs/GS and siRNA@FA‐EVs/GS groups compared with the IUA group. Therefore, consistent with the in vitro results, this study demonstrated that endometrial injury induced STAT1 excessive phosphorylation and macrophage M1 polarization, consequently promoting pro‐inflammatory cytokines release and transformation of ESCs to myofibroblast (Figure 10E,F). Notably, siRNA@FA‐EVs/GS could reverse these results and break the vicious cycle of “ intrauterine adhesion – separation – re‐adhesion”, emphasizing its role in STAT1‐mediated macrophage M1 polarization and IUA formation. This therapeutic potential of siRNA@FA‐EVs/GS in mitigating IUA is attributed to M1 macrophage targeting, efficient knockdown, and excellent bio‐adhesion properties.

## 3 Conclusion

In conclusion, we developed a hydrogel of siRNA@FA‐EVs/GS for the prevention and treatment of IUA. This hydrogel is injectable and has good adhesive properties and facilitates controlled release of siRNA@FA‐EVs. We found that upon injection this hydrogel into uterine cavity, a protective hydrogel layer formed on the surface of injury sites and sustained release STAT1‐siRNA‐loaded FA‐EVs to curtail M1 macrophages polarization and associated pro‐inflammatory factor release through inhibiting STAT1 phosphorylation, resulting in reduction of myofibroblasts activation and collagen deposition to prevent the formation of IUA, promote functional endometrial regeneration and restore fertility. Thus, this topical administration of siRNA@FA‐EVs/GS hydrogel represents a promising approach to reducing the incidence of IUA in the clinical setting.

## 4 Experimental Section

### Materials

Female Sprague‐Dawley (SD) rats aged 8–10 weeks weighing 220–250 g were purchased from Shanghai Laboratory Animal Company (Shanghai, China). The RAW264.7, C166, murine‐derived endometrial stromal cells (ESC) endometrial epithelial cells, and human‐derived bone marrow mesenchymal stromal cells (BMSCs) were sourced from Procell (Wuhan, China). The snail mucus (SM) was purchased from Xinyuhong Biomedical Technology Company (AL44946371544, Hubei, China). Folic acid (FA), 1‐(3‐dimethylaminopropyl)‐3‐ethylcarbodiimide hydrochloride (EDCI), 1,2‐distearoyl‐sn‐glycero‐3‐phosphoethanolamine‐N‐[amino(polyethylene glycol)‐2000] (DSPE‐PEG‐NH_2_) and Lipopolysaccharide (LPS) were bought from MedChemExpress (NJ, USA). Methacrylate gelatin (GelMA, EFL‐GM‐60, 60% graft degree) and lithium phenyl‐2,4,6‐trimethylbenzoylphosphinate (LAP) were supplied by EFL (Jiangsu, China). Culture medium and fetal bovine serum (FBS, SA112.02) used for cell culture were purchased from CellMax (Gansu, China). Transwell plates were obtained from NEST Biotechnology. All RT‐PCR primers were synthesized by Sangon Biotech. Co. Ltd. (Shanghai, China). Carboxyfluorescein (FAM) labeled STAT1‐siRNA was synthesized by GenePharma Co. Ltd. (Shanghai, China). Total RNA Extraction Kit was bought from Beijing Boxbio Science & Technology Co., Ltd. Taq Pro Universal SYBR qPCR Master Mix was purchased from Vazyme Biotech Co., Ltd (Nanjing, China). The enzyme‐linked immunosorbent assay (ELISA) kits for tumor necrosis factor‐α (TNF‐α) and interleukin‐1 beta (IL‐1β) were purchased from NeoBioscience Technology Co. Ltd (Shenzhen, China), and for collagen (Col) III and fibronectin (FN1) were bought from AMEKO (Lianshuo Technology Co. Ltd, Zhejiang, China) and CUSABIO (CSB‐E04553r, https://www.cusabio.com/, Hubei, China). The TRITC‐phalloidin reagent was obtained from Solarbio (Beijing, China). Cell Counting Kit‐8 was purchased from Life‐iLab (AC11L054, Shanghai, China). EVs were labeled by 3,3′‐dioctadecyloxacarbocyanine perchlorate (Dio, US EVERBRIGHT, Suzhou, China). The rabbit derived α‐SMA, CD68, Col III, and FN1 primary antibodies were bought from Cell Signaling Technology (Danvers, MA, USA), ER, and PR antibodies were obtained from Absin (Shanghai, China), and β‐actin primary antibody was purchased from TransGen Biotech, China. The anti‐phosphorylated STAT1 (pSTAT1) and anti‐CD86 were bought from HUABIO (Hangzhou, China). Sparkjade ECL super was purchased from Shandong Sparkjade Biotechnology Co., Ltd. The antibodies of EVs biomarker (Alix, CD9, TSG101, and Calnexin) were sourced from Abcam (MA, USA). All antibodies mentioned above were diluted in NCM universal antibody diluent from New Cell & Molecular Biotech (Jiangsu, China).

### Multi‐omics Analysis and RNA Sequence (RNAseq)

To clarify the key genes of endometrial macrophages between normal tissues and IUA, IUA proteomics published by Ye et al and the IUA macrophages RNAseq dataset (GSE224288) were collected from Gene Expression Omnibus (GEO) database to perform multi‐omics analysis. For proteomics analysis, differently expressed proteins (DEPs) were screened based on the threshold (fold changes > 1.2 or < 0.83, and p‐value < 0.05) provided by Ye et al.^[^ ^49^ ^]^ For RNAseq analysis, differently expressed genes (DEGs) were identified based on the threshold (fold changes > 2 or < 0.5, and p‐value < 0.05). The two sets of data were visualized in volcano plots and heat maps. Gene set enrichment analysis (GSEA) and GO and KEGG analysis were also performed to elucidate the function of DEPs and DEGs. Subsequently, take the intersection of the two via Venn analysis to obtain 2 key genes (STAT1 and F5). The above analysis was performed through the Sangerbox platform (http://sangerbox.com/Tool). To explore the effect of siRNA@FA‐EVs/GS on intrauterine injury, IUA tissues were collected from injury sites of rats treated with or without siRNA@FA‐EVs/GS hydrogel on 3rd day after surgery to establish a gene dataset. Total RNA extraction from IUA tissues was subjected to ribosomal RNA removal and reverse transcription to obtain first‐strand cDNA. The complementary strand was sequentially fabricated and the single “A” base and adapter were ligated to cDNA fragments. The final cDNA library was created by PCR and pair‐end sequencing was performed on the basis of the Illumina sequencing platform provided by XuRan Biotechnology Co., Ltd. (Shanghai, China). The associated analysis was consistent with the above methods.

### Isolation and Identification of BMSCs‐derived EVs

BMSCs‐derived EVs were extracted by gradient ultracentrifugation according to the guidelines provided by the International Society for Extracellular vesicles.^[^ ^50^ ^]^ When the confluency of BMSCs reached 70–80% under microscopic observation, the culture medium was replaced with EVs‐free complete serum and collected after 48 h. Subsequently, the cell supernatant was centrifuged at 300 × g for 10 min to eliminate cells in a cooling centrifuge, then at 2000 × g for 20 min to eliminate dead cells, and 4 °C 10 000 × g for 30 min to eliminate cell debris. After that, the supernatant was obtained and placed in ultra‐high speed centrifuge tubes at 100 000 × g ultracentrifugation for 70 min. EVs were retained at the bottom of the centrifuge tube and resuspended in sterile phosphate buffer saline (PBS) solution after removing the supernatant. To identify the morphology and biological characterization of EVs, the extracted EVs were fixed in a glutaraldehyde solution, and negative staining with phosphotungstic acid on the copper net was performed. The morphology of EVs were observed by transmission electron microscopy (TEM, Talos F200X G2, Thermo Fisher, USA). The diameters and zeta potential of EVs were measured by dynamic light scattering (DLS) with a Malvern Zetasizer Nano ZS90 (Malvern, UK). Additionally, western blotting was adopted to identify the EVs biomarkers including Alix, CD9, TSG101, and Calnexin.

### Synthesis and Characterization of DSPE‐PEG‐FA

As described in the previous report, the first step in this reaction was to activate the carboxy group of FA by adding pyridine and EDCI into FA/DMSO solution and stirring for 2 h at room temperature.^[^ ^51^ ^]^ Subsequently, DSPE‐PEG‐NH_2_ dissolved in DMSO was added and the reaction mixture was stirred overnight to ensure a complete reaction. After completion of the reaction, pyridine was removed from reaction systems using rotary evaporation. Then, the reaction mixture was dialyzed in a dialysis bag (MWCO = 3000 Da) against double‐distilled water to remove unreacted components and freeze‐dried under a vacuum to obtain DSPE‐PEG‐FA. The product was dissolved in deuterated DMSO and confirmed successful conjugation by ^1^H NMR detection.

### Preparation and Characterization of FA‐modified EVs Loading with STAT1‐siRNA

FA‐modified EVs (FA‐EVs) were fabricated by the self‐assembly method that DSPE‐PEG‐FA was incorporated into the membrane of EVs with the mass ratio of 1:1 for 2 h incubation at 37 °C. Afterward, ultracentrifugation with 120,000 g at 4 °C for 70 min was performed to remove unincorporated DSPE‐PEG‐FA and obtained purified FA‐EVs. To screen the highest interference efficiency of STAT1‐siRNA, three separate STAT1‐siRNA candidates (Table S1, Supporting Information) were designed and transfected into LPS‐induced macrophages to detect the expression of STAT1. Subsequently, FAM‐labeled STAT1‐siRNA loaded FA‐EVs (siRNA@FA‐EVs) were prepared by electroporation. Briefly, 1 µg STAT1‐siRNA and 10 µg FA‐EVs were completely resuspended in an electroporation buffer and performed electroporation at different voltages using Gene Pulser Xcell (Bio‐rad, CA, USA). After electroporation, the siRNA@FA‐EVs were incubated at 37 °C for 30 min to recover the extracellular vesicle membrane and then pipetted from the tubes for further study. TEM, DLS, and western blotting were performed to detect the effects of FA modification and siRNA transfection on the morphology and biological characterization of EVs.

### Efficacy of Cellular Uptake of siRNA@FA‐EVs

Before gene silencing cellular experiments, Raw264.7 macrophages were seeded in 24‐well plates with a density of 10^5^ cells and divided into the M0 subtype group (no treatment), M1 subtype group (induced by 1 µg mL^−1^ LPS for 24 h) and M2 subtype group (induced by the combination of 20 ng mL^−1^ IL‐4 and 10 ng mL^−1^ IL‐13 for 24 h). Then, 10µg Dio‐labeled siRNA@FA‐EVs or siRNA@EVs were added into each group and co‐incubation for 6 h. Subsequently, the EVs‐free medium was discarded and washed with PBS solution and then stained with Hoechst 33342 and phalloidin. Finally, the cellular uptake of Raw264.7 was quantified under the fluorescence microscope observation. In addition, flow cytometry (CytoFLEX S, Beckman, Norway) was also applied to detect the efficacy of cellular uptake in different groups.

### GelMA/Snail Mucus (GS) Hydrogel Preparation and Characterization

GS hydrogel was prepared by the following process with two components including GelMA and Snail mucus. First, GelMA with 60% substitution was dissolved in PBS solution. Then, snail mucus (SM) was added to GelMA solution to fabricate different masses of GS hydrogel. Finally, LAP was added at a final concentration of 0.25% and GS hydrogel were formed through UV curing (405 nm, 30 mW cm^−2^). For adhesive property examination, GelMA/SM‐0% and GelMA/SM‐1%, GelMA/SM‐5% and GelMA/SM‐10% hydrogels were cut rectangles (20 mm × 10 mm, *n =* 3) and uniformly placed between two porcine skin tissues. Subsequently, the force‐displacement curve was evaluated by lap shear test (Instron 5565) and adhesive strength was calculated by dividing the load force by the overlapping area (20 mm × 10 mm). To measure work of adhesion performance in the normal direction, 180° peeling adhesion resistance tests were performed and the overlapping area was 20 mm × 10 mm. The adhesion energy was calculated by 2 × *F*/*w*, where *F* was the plateau force during peeling and *w* was the width of the specimen. The porcine skin was used in the peeling tests and the peeling rate was fixed at 100 mm min^−1^. In addition, an aging hydrogel was fabricated by reciprocating sliding friction using a ball‐on‐disk tribometer (UMT‐TriboLab, USA). A Teflon ball (8 mm) was reciprocated against a stainless‐steel plate with a fixed path length of 3 mm for 20 min at a loaded force of 1 N and a sliding frequency of 4 Hz. The rheological properties of the aged hydrogels were also tested using a rheometer (TA Instruments, USA). A compression test was performed on a mechanical tester (Instron, USA). The specimens (30 mm × 10 mm × 3 mm) were stretched to a maximum strain of 50% for 100 cycles. For the compression test, cylindrical samples (*d* = 10 mm, *h* = 5 mm) were prepared and tested. To evaluate the self‐healing capability of this hydrogel, hydrogels were stained with red and blue pigments. After gelation, two hydrogel pieces were placed adjacent to each other in a plastic dish. Covered and kept in a cell incubator for 10 min, the self‐healing ability was evaluated. After 10 min, the healed hydrogel was pulled at both ends using tweezers for observation. The lyophilized hydrogels were separately immersed in excess water until completely dissolved and the maximum water absorption of the gel was carried out by weight difference. Next, the hydrogel was moved to a fume hood and weighed at the indicated time and the water loss curve of the hydrogel was plotted. The optimal hydrogel was selected for further experiments based on adhesion properties. The chemical composition variation between GelMA and SM were detected by Fourier transform infrared spectroscopy (FTIR) assay. Lyophilized GelMA, SM and GS were respectively ground into powders and then mixed with KBr pellet to record its spectrum using an FTIR spectrometer (Nicolet Avtar 360, USA). The topological microstructures of freeze‐dried hydrogels were recorded using a scanning electron microscope (SEM, Sirion 200, FEI, USA). To test the injectability of each hydrogel, a 1 mL syringe with the needle was used to inject hydrogel into the uterine cavity under UV light, then hydrogel was taken out from the exposed uterine cavity and recorded by a digital camera.

### Controlled Release of siRNA@FA‐EVs/GS Hydrogel

siRNA@FA‐EVs/GS hydrogel was prepared by adding 20 µg siRNA@FA‐EVs into 5 mL GS solution before UV curing. To detect the controlled release property, siRNA@FA‐EVs/GS hydrogel was transferred into tubes with 20 mL of PBS solution at 37 °C. At different points (0, 6, 12, 24, 48, 72, 96, 120, 144, and 168 h), 1 mL supernatant was obtained, and equal PBS solutions were replenished. EVs were labeled with Dio, and therefore the concentration of siRNA@FA‐EVs was quantified by measuring the fluorescence of the removed supernatant. In addition, After the hydrogel was equilibrated, the hydrogel was removed and weighed at this point (W0). Then, the hydrogel was taken out and weighed at each point and the mass was recorded as Wt. The degradation rate of the hydrogel was calculated by the formula. The experiment was repeated in 3 groups. Degradation rate (%) = (W0‐Wt) / W0 × 100%.

The fluorescent motif FAM in the siRNA was labeled and the release of siRNA by detecting the fluorescence intensity was measured. siRNA@FA‐EVs/GS hydrogel was transferred into tubes with 20 mL of PBS solution at 37 °C. At different points (0, 6, 12, 24, 48, 72, 96, 120, 144, and 168 h), 1 mL supernatant was obtained, and equal PBS solutions were replenished. After obtaining the standard curve of FAM‐siRNA, supernatant at different time points were transferred to a 96‐well plate and analyzed via a microplate reader at 488 nm to measure fluorescence intensity. Then the cumulative release of STAT1‐siRNAs was calculated according to the standard curve of FAM‐siRNA.

### In Vitro Cytocompatibility Evaluation

CCK‐8 and dead/live assay were applied to evaluate the cytocompatibility of siRNA@FA‐EVs/GS hydrogel. Briefly, RAW267.4 were seeded in the lower chamber of transwell plates with the appropriate density and then incubated with the CCK‐8 solution or calcein AM/ propidium iodide (PI) solution. For the CCK‐8 assay, the optical density of the supernatant at 450 nm was measured by a microplate reader. For the dead/live assay, fluorescence microscopy observation indicated that living cells appeared green fluorescence, while dead cells appeared red fluorescence. The six areas were randomly selected and calculated the apoptosis rate of RAW267.4.

### Cells Culture

Transwell plates were used to detect the effect of siRNA@FA‐EVs/GS on macrophage polarization. Briefly, siRNA@FA‐EVs/GS was uniformly applied to the upper chamber and RAW264.7 cells were cultured in the lower chamber with free‐EVs medium in the constant‐temperature incubator. To mimic in vivo inflammatory microenvironment, 100 ng mL^−1^ LPS was added into plates when the cell confluence reached 70% and incubated for 16h. The Raw264.7 macrophages were randomly divided into five groups: without any treatment group (Control), LPS treatment group (LPS), LPS and GS hydrogel treatment group (LPS+GS), LPS and siRNA@EVs/GS hydrogel treatment group (LPS+siRNA@EVs/GS) and LPS and siRNA@FA‐EVs/GS hydrogel treatment group (LPS+siRNA@FA‐EVs/GS).

To examine the interaction between macrophage‐derived inflammatory cytokines and myofibroblast transformation of ESCs, Macrophage supernatants containing LPS were removed, and then the supernatants were collected after adding a complete medium and culture for 24h. ESCs were seeded in 24‐well plates in complete medium and replaced with treated‐macrophage supernatants after attaining appropriate confluency, and cultured for 24 h for further experiments.

### Intrauterine Adhesion (IUA) Model

The surgical procedures and animal care conditions were approved by the Institutional Animal Care Committee of Tongji University and fully consistent with the institutional guidelines. Consistent with previous IUA model establishment methods, mechanical scratching was performed at the endometrium, and detailed procedures were indicated in Figure S12 (Supporting Information).^[^ ^52^ ^]^ Briefly, all surgical instruments and supplies should be autoclaved before operation and subsequent surgical operation was performed in a strictly sterile environment. Rats were anesthetized by intraperitoneal injection of 2% sodium pentobarbital (0.3 mL/100 g). After the deep anesthesia, the lower abdomen was sterilized and incised with a 4 cm longitudinal incision to expose both sides of the uterus. A 2 mm diameter incision was made on both sides of the uterus near the cervix, and then the uterine cavity was inserted and scraped until the endometrium appeared obvious congestion and became rough. The hydrogel was injected into the uterine cavity but not used in the IUA group. Subsequently, the uterus and abdomen were sutured closed layer by layer directly using 6‐0 absorbable sutures. After the operation, rats were placed in a warm cage to maintain their body temperature until they regained consciousness from euthanasia. The rats were sacrificed at 3 and 28 days after the operation and uterine samples were obtained. Forty rats with regular estrous cycles were involved in this study and divided into four groups randomly, including the IUA model group, IUA model treated with GS hydrogel (IUA+GS), siRNA@EVs/GS hydrogel (IUA+siRNA@EVs/GS) and siRNA@FA‐EVs/GS hydrogel (IUA+siRNA@FA‐EVs/GS). Each group at different points included 6 rats for further experiments (*n* = 6).

### Gross Observation and Histological Evaluation

After the operation, gross observation of the uterus was performed based on the following aspects: uterine shape, congestion degree, surface roughness, and tissue swelling. For histological evaluation, uterus segments were fixed in 4% paraformaldehyde, dehydrated in the gradient concentration of alcohol, and embedded in paraffin. Hematoxylin/Eosin (HE) and Masson's trichrome staining were adopted to evaluate endometrial thickness, the number of glands, and the percentage of fibrosis areas using image J software.

### Number of Embryos Test and Live Birth Test

Female rats of each group were mated with male rats at 28 days post‐surgery, and successful mating was confirmed by vaginal smear. The rats were euthanized at mid‐to‐late gestation and uteri were collected to analyse the number of embryos. Meanwhile, the number of live births in each group of rats were observed and the weights of the offspring were measured. Pups in each group were nurtured for an additional 14 days to observe the development of the offspring.

### Immunohistochemical and Immunofluorescence Analysis

After fixation, tissues or cell slides were permeabilized with 0.5% Triton X‐100 solution, blocked with 5% goat serum, and then immersed with primary antibodies (CD68, CD86, Arg1, pSTAT1, α‐SMA, Col III, FN1, ER and PR) at 4 °C overnight. After washing to remove unbound antibodies by PBS solution, horseradish peroxidase‐labeled or fluorescently labeled secondary antibodies were evenly covered onto slides and incubated for 1 h at 37 °C. The protein expression of Col III, FN1, ER and PR was visualized by DAB staining. The nuclei were stained with hematoxylin counterstaining or DAPI labeling. Finally, six random fields of view were acquired under the DMi8 fluorescence microscope from Leica Microsystems LTD. Image‐Pro Plus was applied to quantify the percentage of positive cells and fluorescence intensity of protein.

### Real‐Time PCR (RT‐PCR)

Total RNA from IUA tissues and cells was extracted and reversely transcribed into complementary DNA. Then, the reaction system was prepared following the manufacturer's instructions and run on the ABI PRISM 7500 Sequence Detection System (Applied Biosystems Inc.). The accumulation of PCR products was recorded in real‐time using the SYBR Green method and the cycle threshold (CT) values were obtained to evaluate the relative mRNA expression through normalization to GAPDH and 2^−ΔΔCT^ methods. The sequences of upstream and downstream primers are shown in Table S2 (Supporting Information).

### ELISA

ELISA detection was applied to quantify the concentrations of pro‐inflammatory and adhesion‐related cytokines (IL‐1β, TNF‐α, Col III, and FN1) in IUA tissues and supernatant. Total protein concentrations in each sample were analyzed by BCA quantification and adjusted to be consistent. Subsequently, the standard calibration curves of relative proteins were drawn on the basis of the absorbances and concentrations of standards at different dilution ratios. Protein samples were added into antibody‐coated microtiter plates and incubated for 2 h at room temperature to ensure specific binding between antibody and target protein. After washing, the samples were immersed in avidin‐biotin‐peroxidase complex for 30 min and tetramethylbenzidine was applied for color development. The optical density of each sample was recorded by the microplate reader and calculated the concentrations according to the standard curve.

### Western Blot

Protein samples were extracted from IUA tissues of different groups and EVs using RIPA lysis solution and detected by BCA protein assay to quantify the protein concentration. Then, protein samples were homogenized using 5X SDS loading buffer and boiled at 100 °C for 5 min to completely denature the proteins. Different molecular weights of protein were separated by SDS–PAGE gels under constant voltage conditions at 100 V and transferred to PVDF membranes at a constant current of 300 mA. The membranes were sequentially blocked by 5% BSA solution, followed by incubation of primary antibodies overnight at 4 °C (p‐STAT1, CD86, Alix, CD9, TSG101, Calnexin, and β‐actin) and secondary antibodies for 1 h at room temperature. Subsequently, the protein bands at the membranes were captured ChemiDoc Touch system (Bio‐rad, CA, USA).

### Flow Cytometry

For cellular uptake and protein expression of Raw264.7 treated with siRNA@FA‐EVs/GS, 1 × 105 cells were collected from plates and labeled fluorescently labeled antibody for 30 min. The percentage of positive cells, uptake efficiency, and phosphorylation level of pSTAT1 were evaluated by CytoFLEX S (Beckman, Norway) and visualized by FlowJo software.

### Statistical Analysis

Data were expressed as mean ± standard deviation (SD). The sample number (*n*) in each experiment represented the number of independent biological samples. SPSS 21.0 statistical software was applied to handle the data. Comparison between different groups were performed through the unpaired Student's t‐test or one‐way analysis of variance (ANOVA), following Tukey's test. The threshold of the statistically significant difference was set as p‐value <0.05.

## Conflict of Interest

The authors declare no conflict of interest.

## Supporting information



Supporting Information

## Data Availability

The data that support the findings of this study are available from the corresponding author upon reasonable request.
