# Supplementary material for: Gene Therapy for Inflammatory Cascade in Intrauterine Injury with Engineered Extracellular Vesicles Hybrid Snail Mucus‐enhanced Adhesive Hydrogels
Source: Adv Sci (Weinh). 2024 Oct 25;12(1):2410769. doi: 10.1002/advs.202410769 (PMC11714243; doi:10.1002/advs.202410769)
Supplement: Supplementary file 1 — Supporting Information [file ADVS-12-2410769-s001.docx]

**Supplementary Files**

**Gene therapy for inflammatory cascade in intrauterine injury with engineered extracellular vesicles hybrid snail mucus-enhanced adhesive hydrogels**

Xiaotong Peng^1^, Tao Wang^1^, Bo Dai^4^, Yiping Zhu^1^, Mei Ji^1^, Pusheng Yang^1^, Jiaxin Zhang^1^, Wenwen Liu^1^, Yaxin Miao^1^, Yonghang Liu^3*^, Shuo Wang^2*^, Jing Sun^1*^

1. Department of Gynecology, Shanghai Key Laboratory of Maternal Fetal Medicine, Shanghai Institute of Maternal-Fetal Medicine and Gynecologic Oncology, Shanghai First Maternity and Infant Hospital, School of Medicine, Tongji University, Shanghai 200092, China

2. Department of Orthopaedics, Shanghai Sixth People’s Hospital Affiliated to Shanghai Jiao Tong University School of Medicine, Shanghai, 200233, China

3. School of Pharmacy and State Key Laboratory of Quality Research in Chinese Medicine, Macau University of Science and Technology, Macao, China

4. Department of Hematology, Huashan Hospital, Fudan University, Shanghai, 200040, China

* Corresponding author.

mail address:

Jing Sun, sunjing61867@tongji.edu.cn;

Shuo Wang, shuowang97@126.com;

Yonghang Liu, yonghangliu@163.com


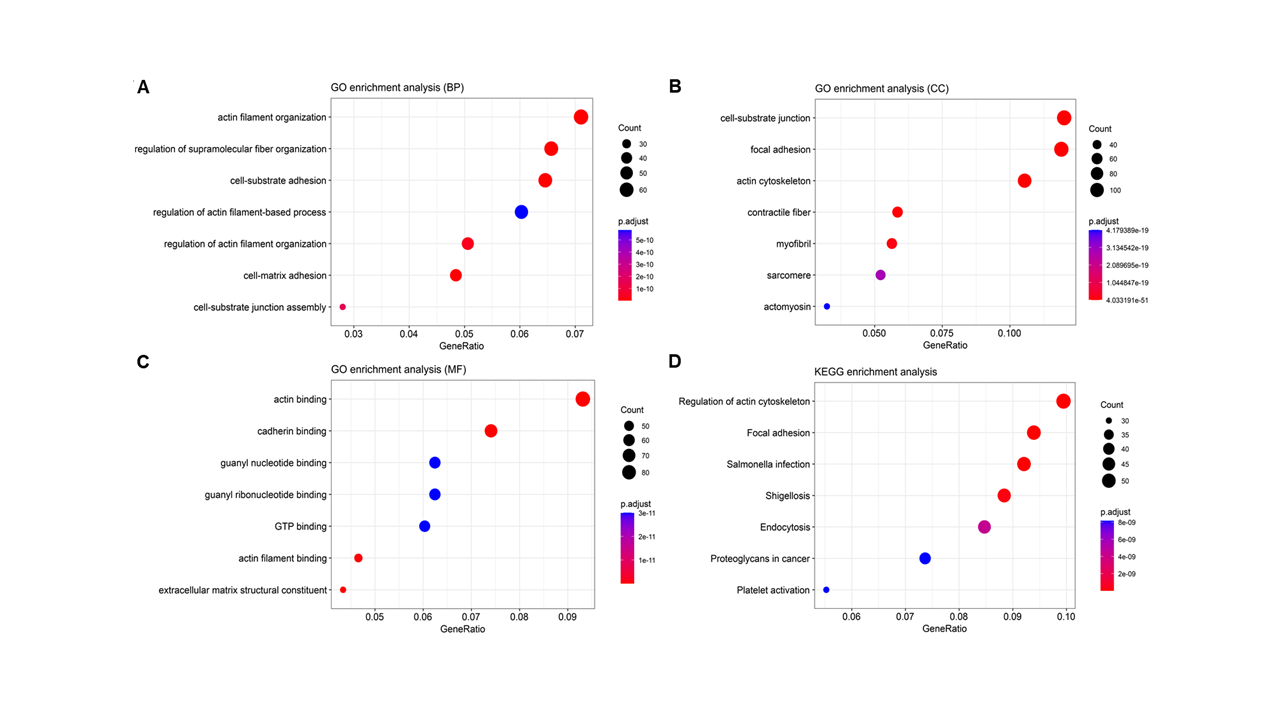


**Figure S1.** KEGG and GO analysis of upregulated DEPs, including A) Biological process (BP), B) Cell composition (CC), C) Molecular function (MF) and D) KEGG.


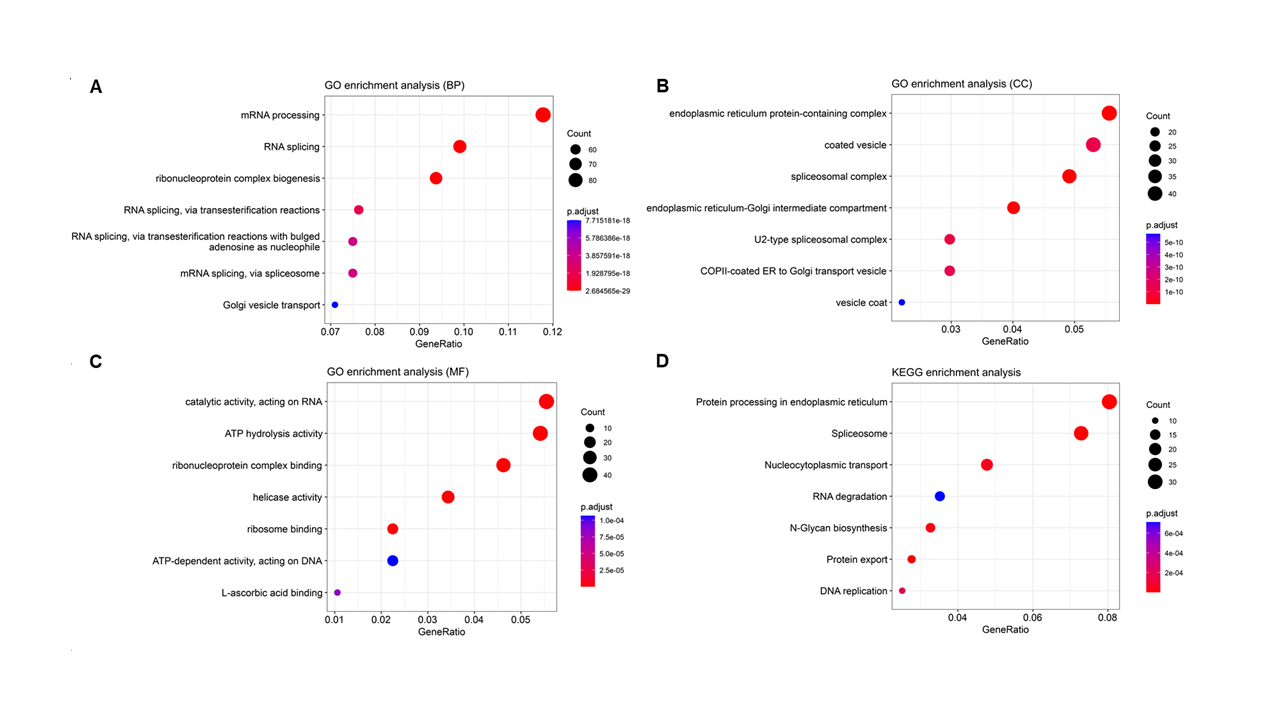


**Figure S2.** KEGG and GO analysis of downregulated DEPs, including A) BP, B) CC, C) MF and D) KEGG.


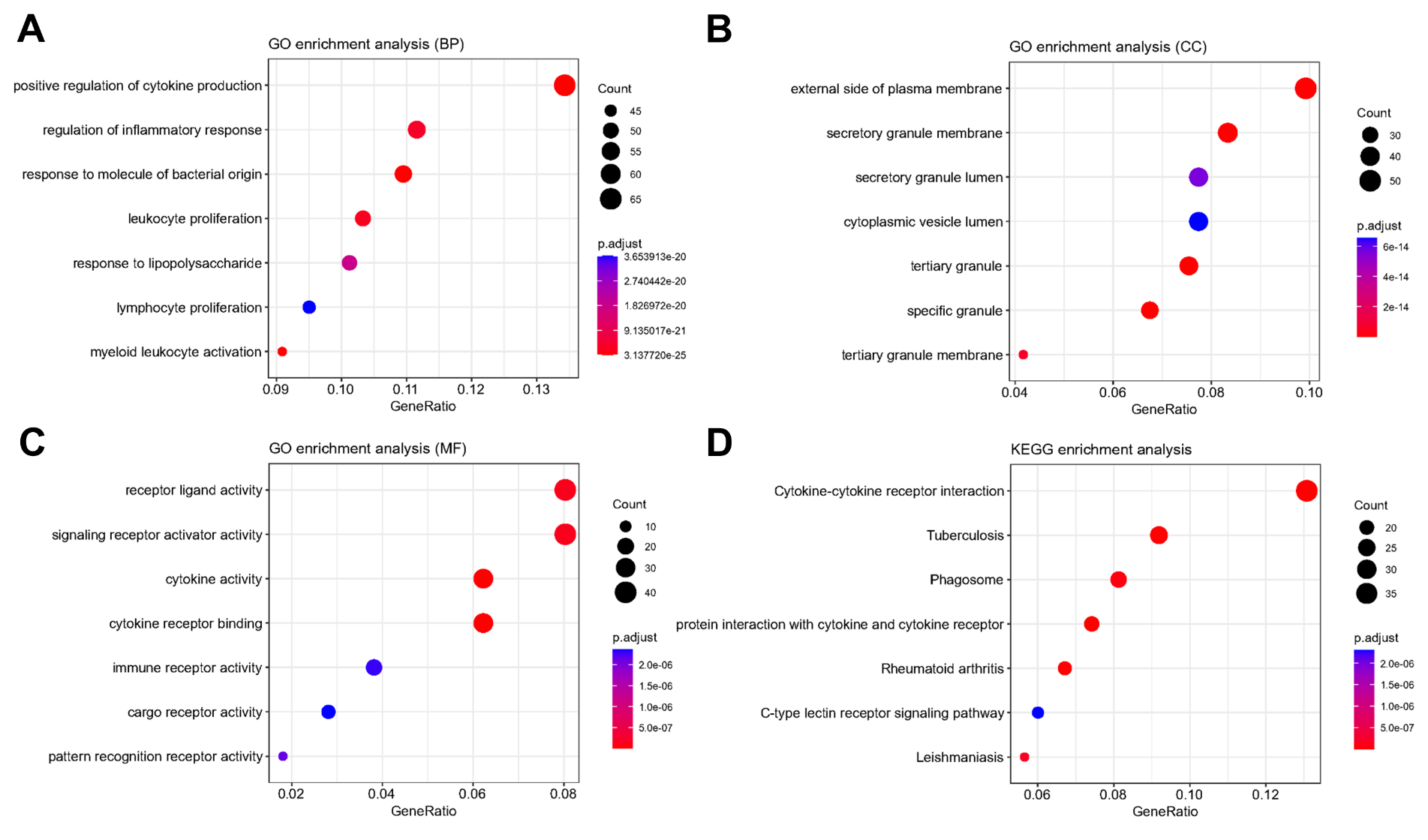


**Figure S3.** KEGG and GO analysis of upregulated DEGs, including A) BP, B) CC, C) MF and D) KEGG.


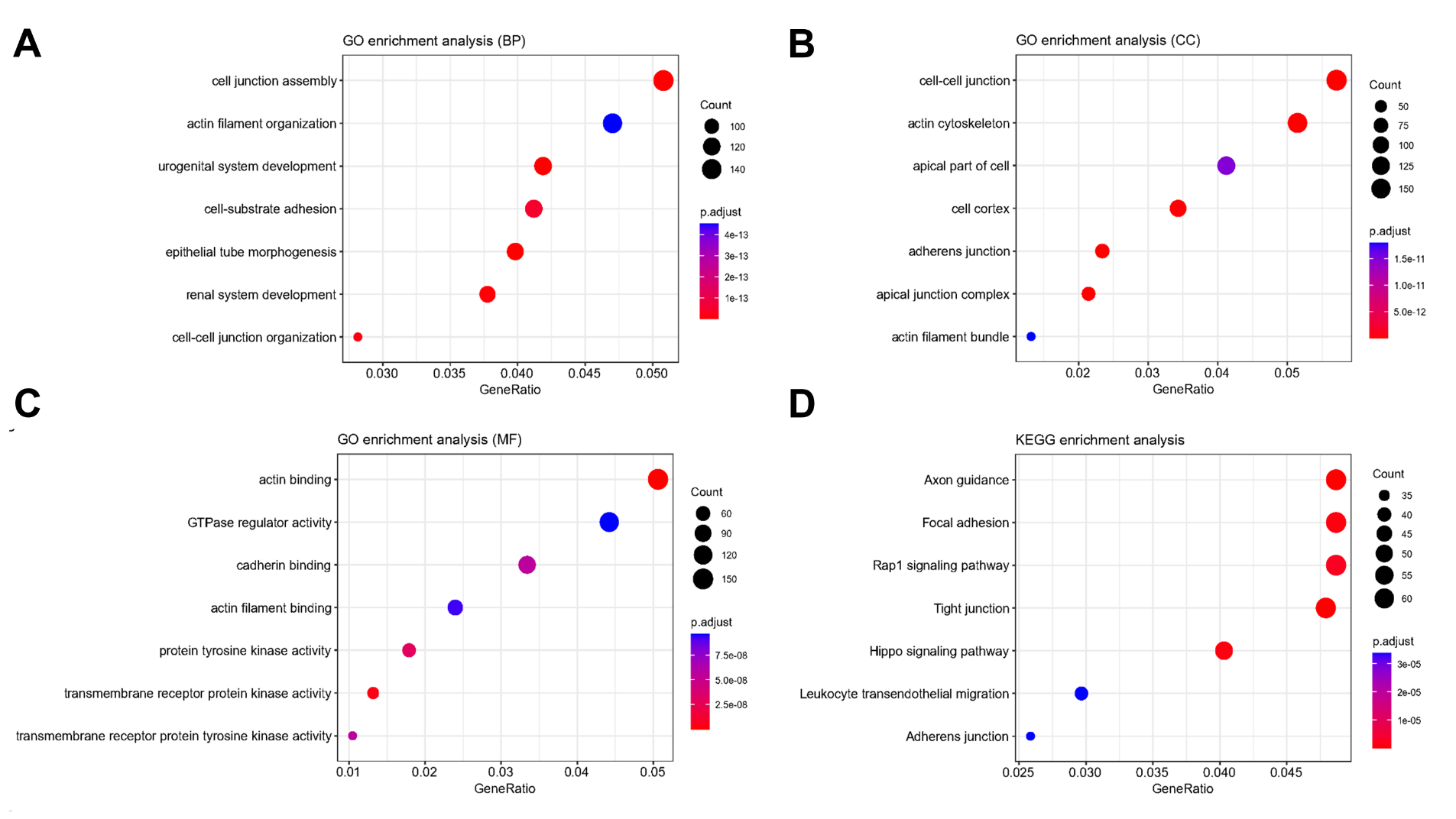
**Figure S4.** KEGG and GO analysis of downregulated DEGs, including A) BP, B) CC, C) MF and D) KEGG.


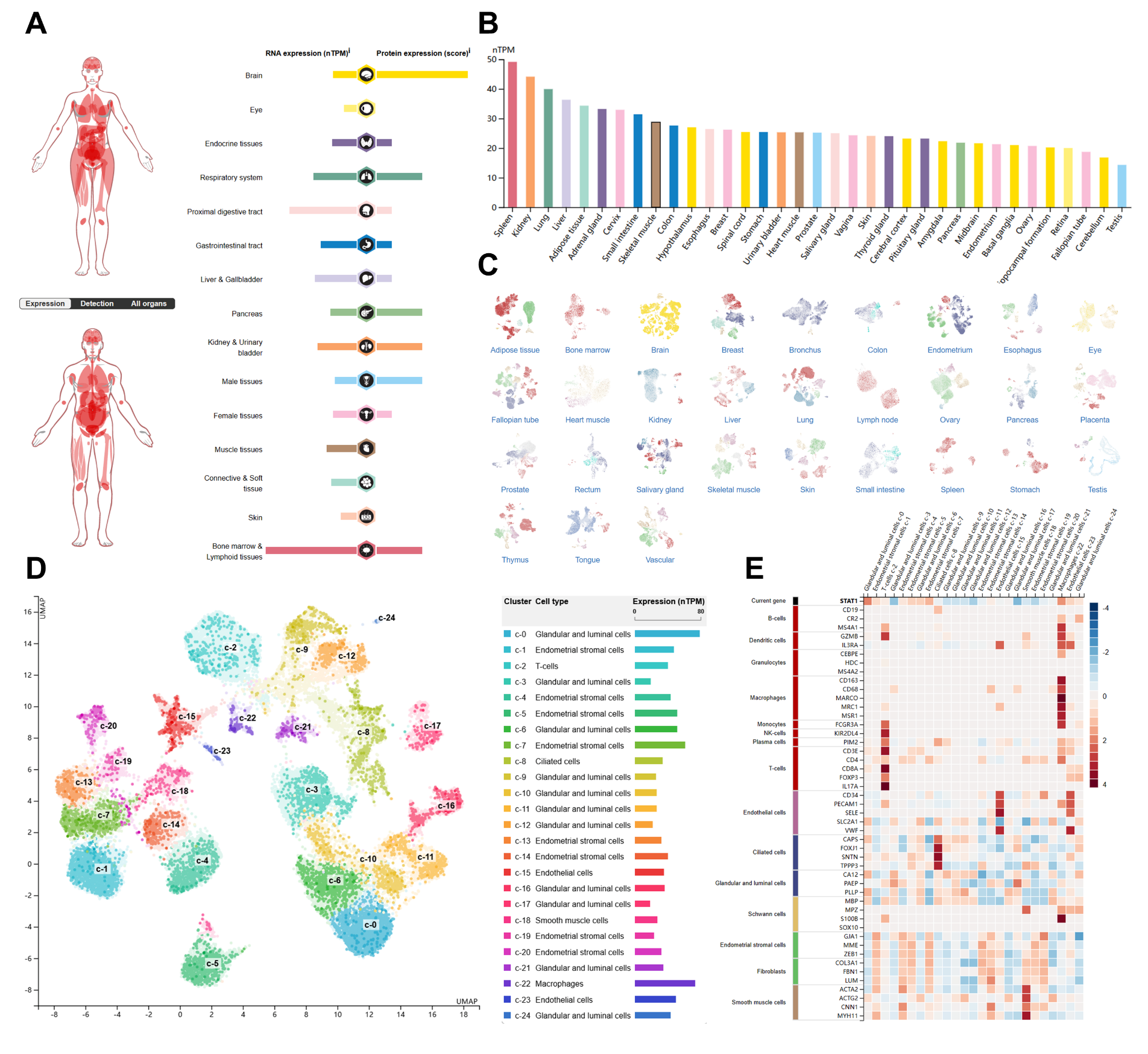


**Figure S5.** A-B) Distribution of STAT1 expression in human organs and tissues; C) Cellular subpopulation distribution of STAT1 in single-cell sequencing of human organs; D) Distribution of STAT1 expression in human endometrium through single cell analysis; E) STAT1 is mainly distributed to macrophages glandular and luminal cells in endometrium.


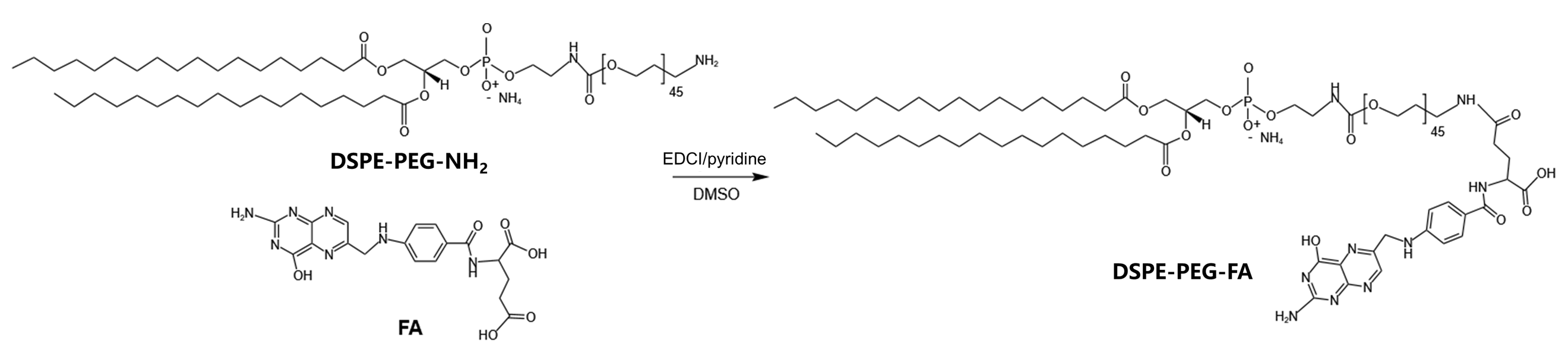


**Figure S6.** Preparation and synthesis scheme of DSPE-PEG-FA.





**Figure S7.** The screening of STAT1-siRNA by RT-PCR. Data are presented as mean ± SD and analyzed by one-way ANOVA, Tukey’s post hoc test. ^&^p < 0.05 compared with control group; ^*^p < 0.05 compared with LPS+STAT1-siRNA1, LPS+STAT1-siRNA2, LPS+STAT1-siRNA3 group, n = 6.


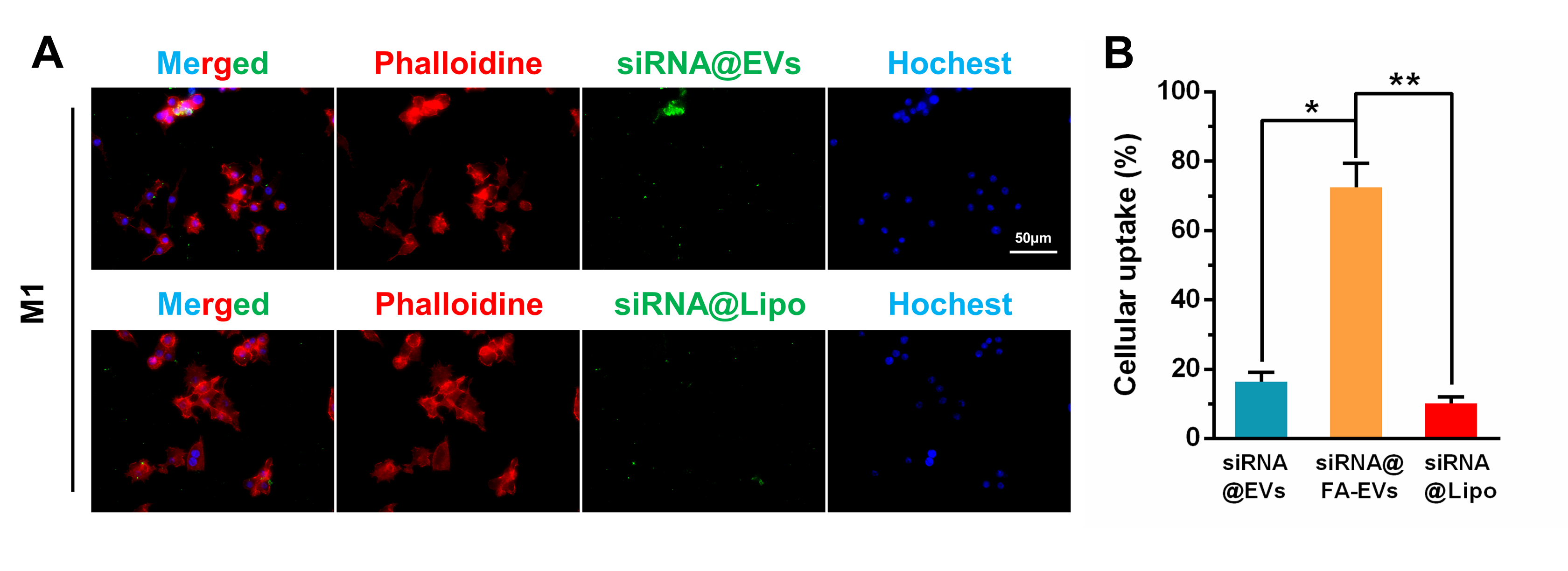


**Figure S8.** A, B) Fluorescence images and cellular uptake of M1 subtype macrophage treated with siRNA@EVs and siRNA@Lipo. *p < 0.05 compared with siRNA@EVs group, **p<0.05 compared with siRNA@Lipo group, n = 6.


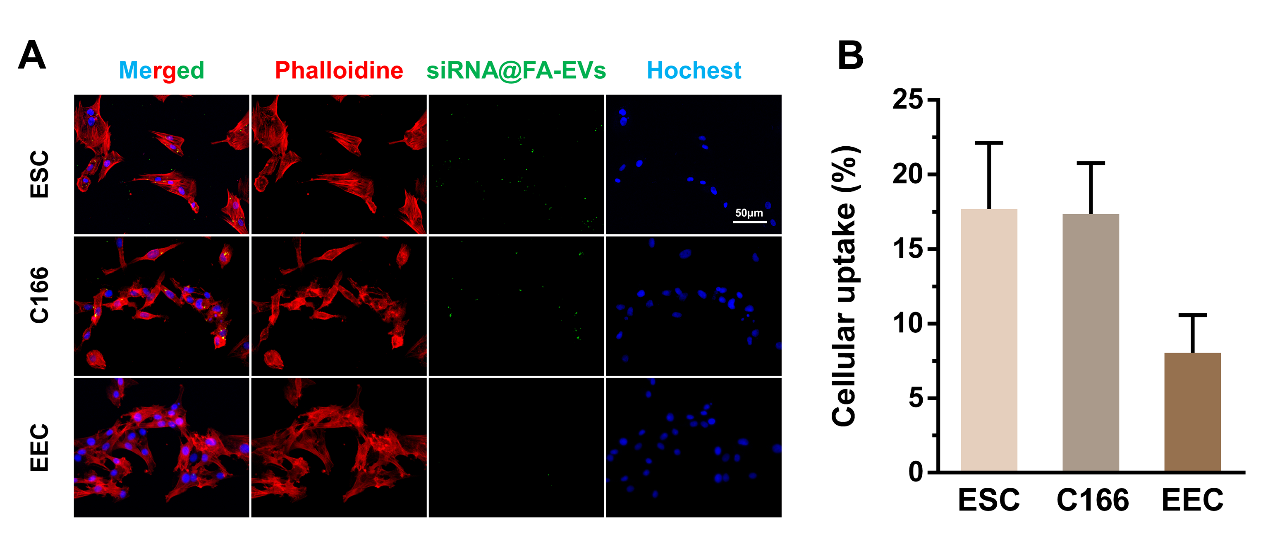


**Figure S9.** A, B) Fluorescence images and cellular uptake of ESC, C166 and EEC treated with FA-EVs.


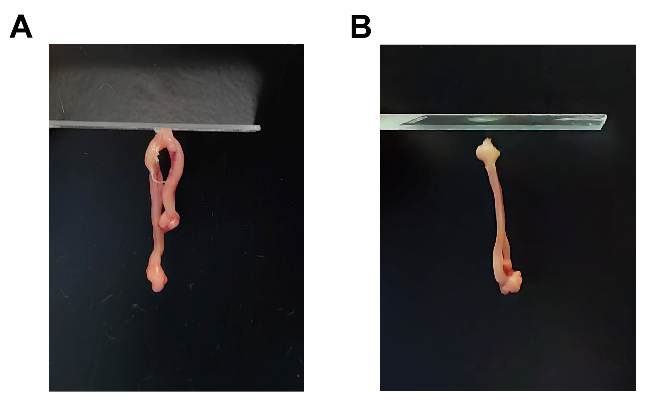


**Figure S10.** Visual experiment for bio-adhesion property of siRNA@FA-EVs/GS (A) and GelMA (B) for uterus.


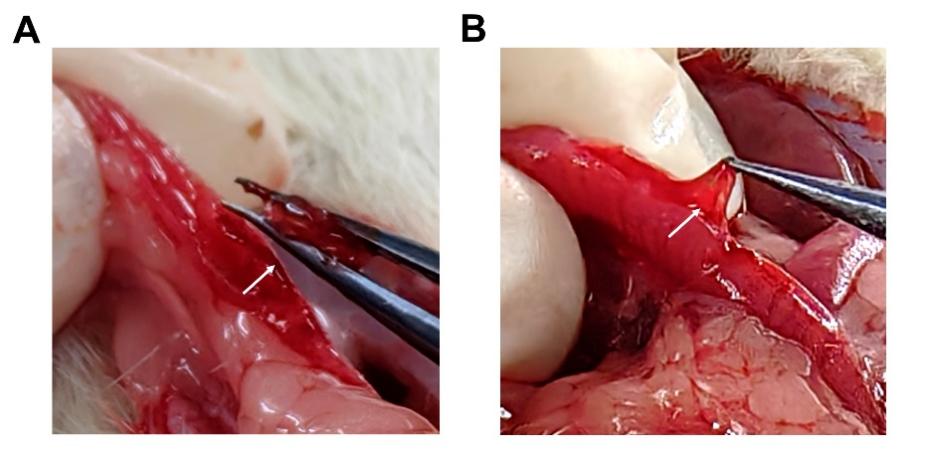


**Figure S11.** The structural integrity and bio-adhesion properties of GelMA (A) and siRNA@FA-EVs/GS (B) hydrogel in the uterine cavity at 3rd day postoperatively.





**Figure S12.** Quantification of STAT1-siRNA release from siRNA@FA-EVs/GS hydrogel measured by FAM fluorescence intensity.





**Figure S13.** The degradation rates of GS hydrogel. Data are presented as mean ± SD, n =3.


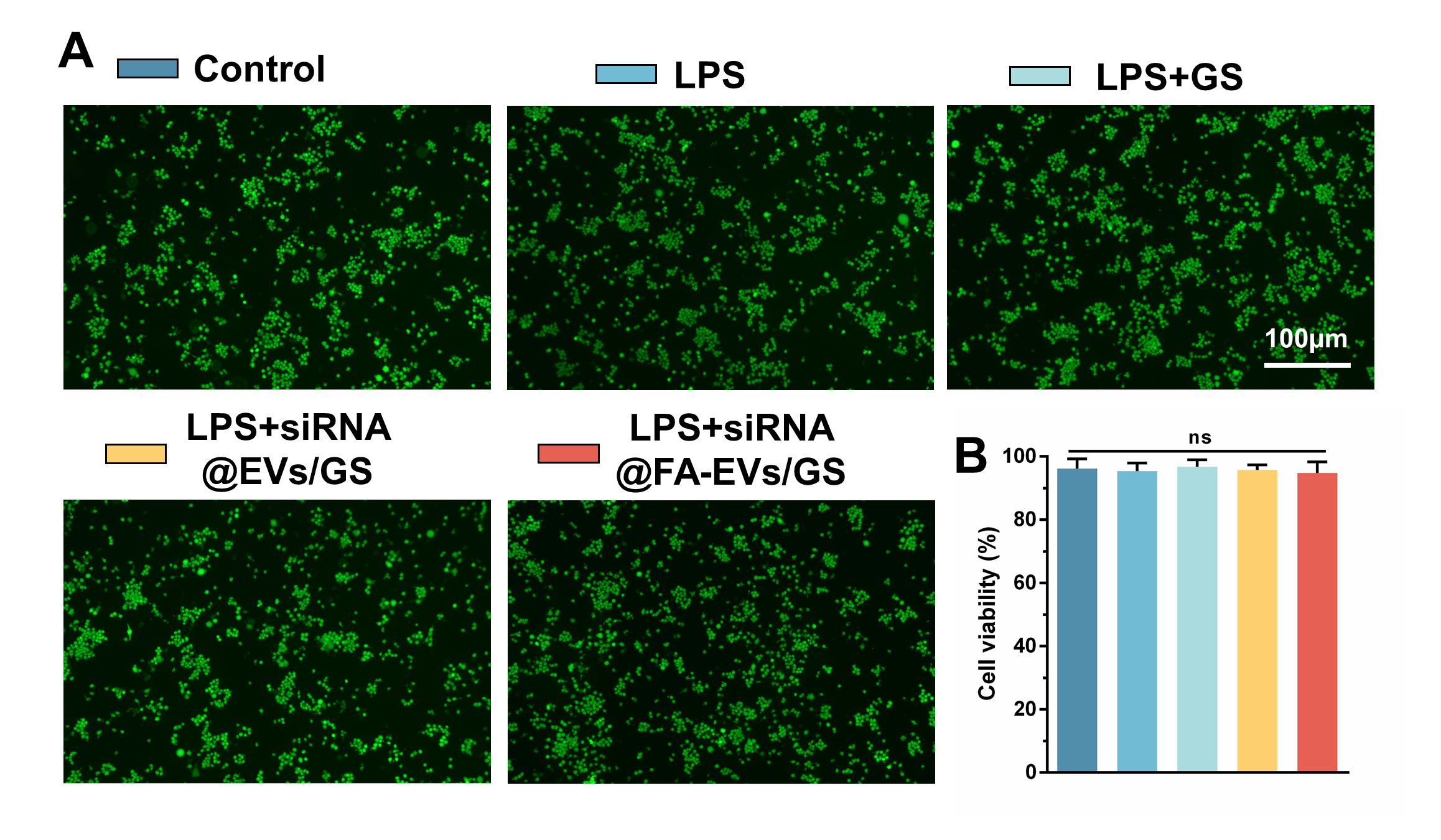


**Figure S14.** Live/Dead analysis of macrophages with different treatment under LPS-induced inflammatory microenvironment. A) Live/Dead images of each group; B) Quantitative histogram of apoptosis rate that was computed as “Dead cells”/ (“live cells” + “Dead cells”). Data are presented as mean ± SD and analyzed by one-way ANOVA, Tukey’s post hoc test. ns, no significance between two groups, n = 6.


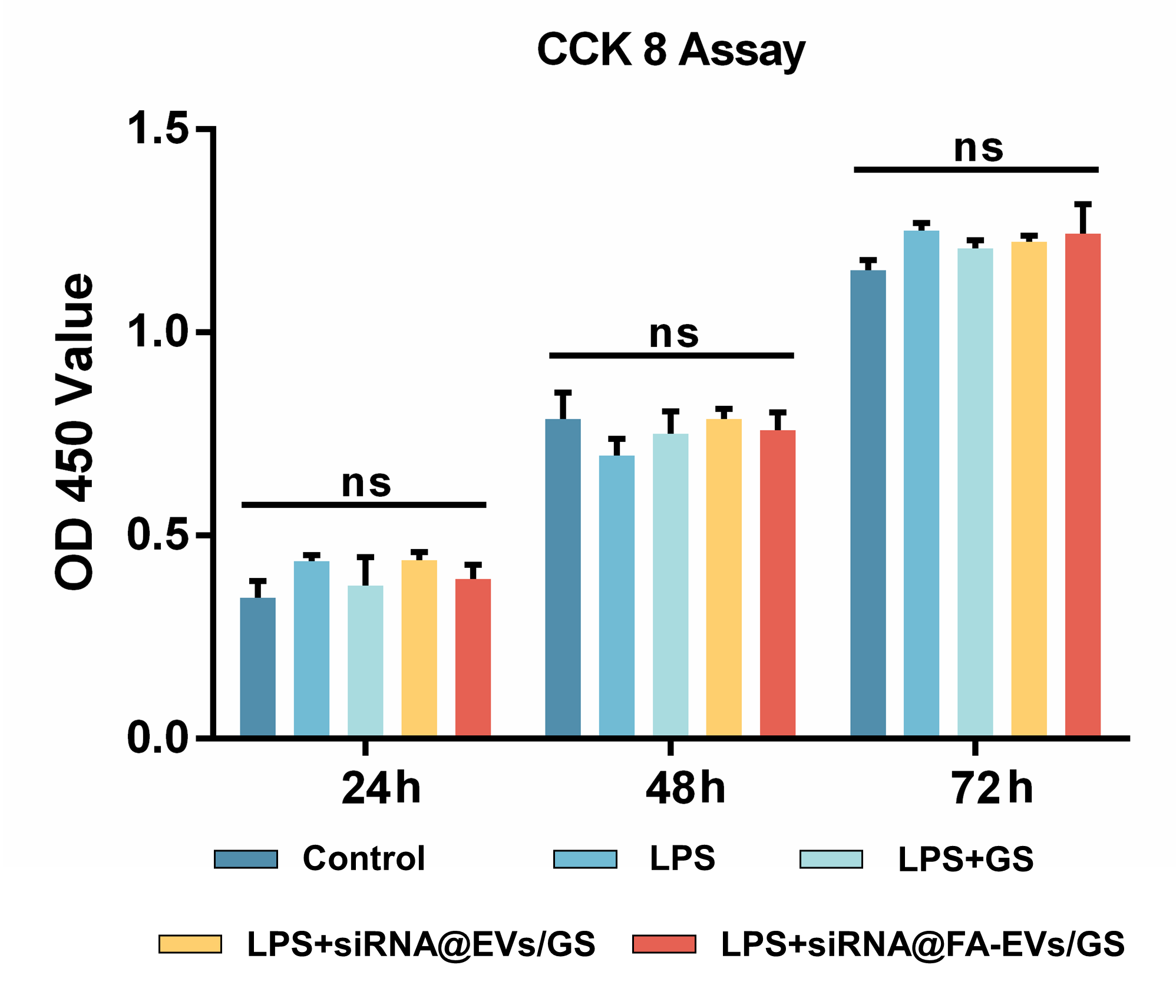


**Figure S15.** CCK-8 analysis of macrophages with different treatment under LPS-induced inflammatory microenvironment. Data are presented as mean ± SD and analyzed by one-way ANOVA, Tukey’s post hoc test. ns, no significance between two groups, n = 6.


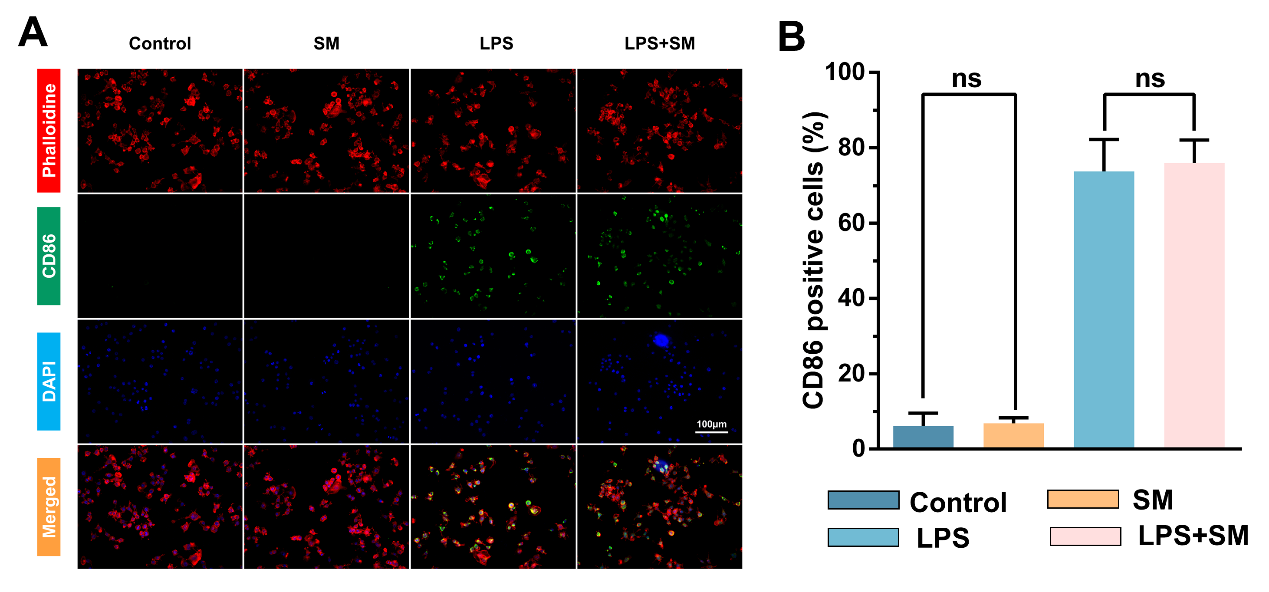


**Figure S16.** A, B) The effect of SM on macrophage polarization under the microenvironment with or without LPS. ns, no significance between two groups, n = 6.


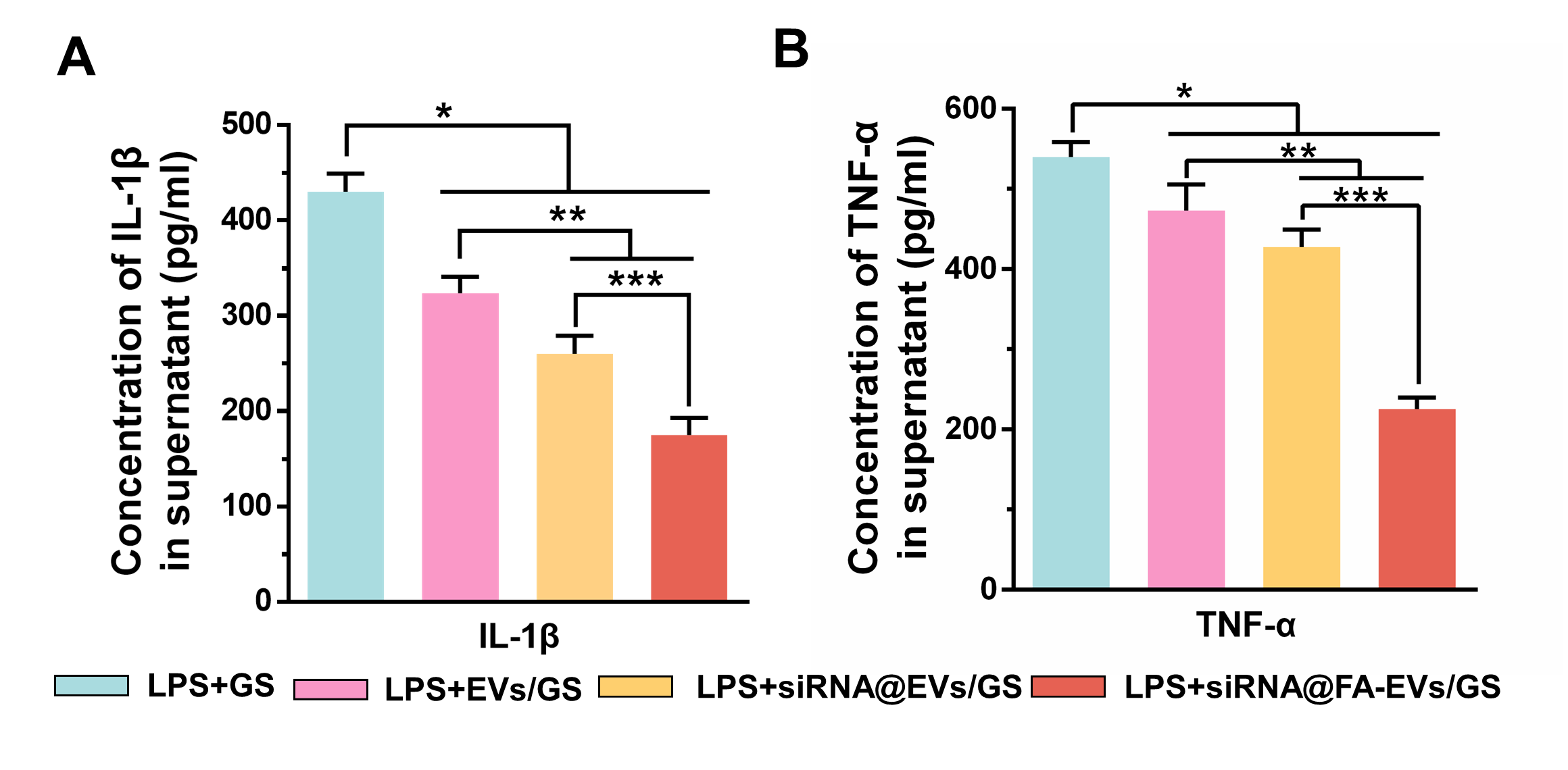


**Figure S17.** A, B) The concentrations of IL-1β and TNF-α in cell supernatant were measured by ELISA. Data are presented as mean ± SD. *p < 0.05 compared to the LPS+GS group, **p < 0.05 compared to LPS+EVs/GS group, ***p < 0.05 compared to the LPS+siRNA@EVs/GS group.


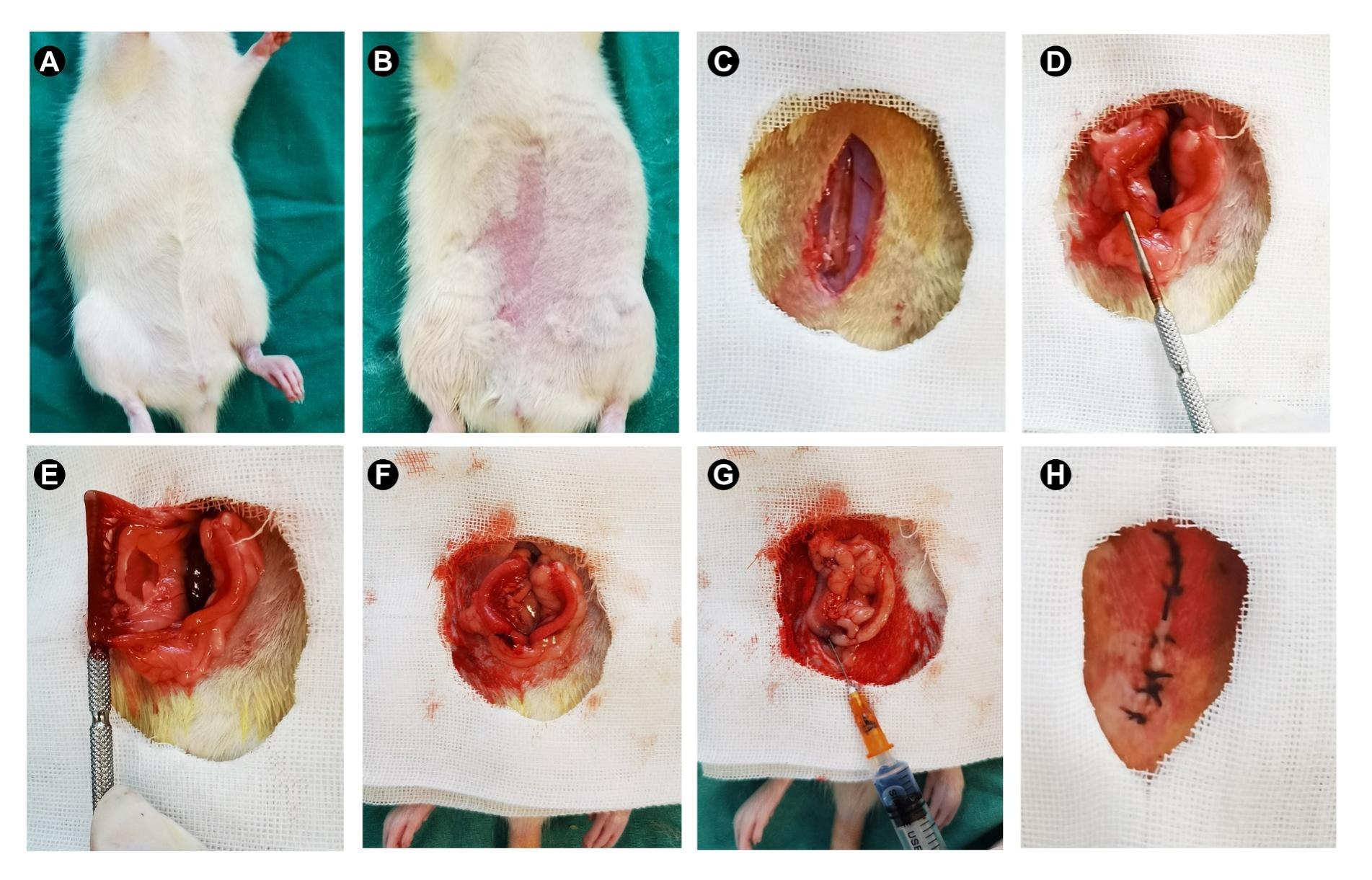


**Figure S18.** A-H) The procedure of rat model of IUA.


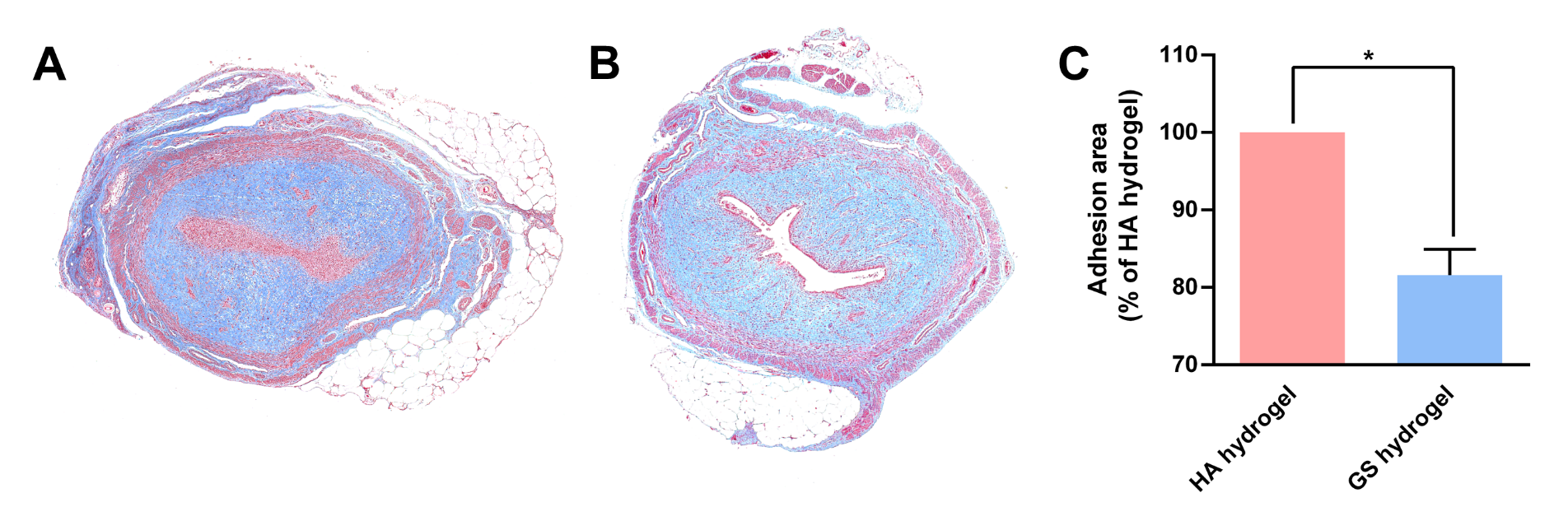


**Figure S19.** A) The Masson staining image of HA hydrogel group. B) The Masson staining image of GS hydrogel group. C) Adhesion area were calculated. Data are presented as mean ± SD. *p < 0.05 compared with HA hydrogel group, n = 6.


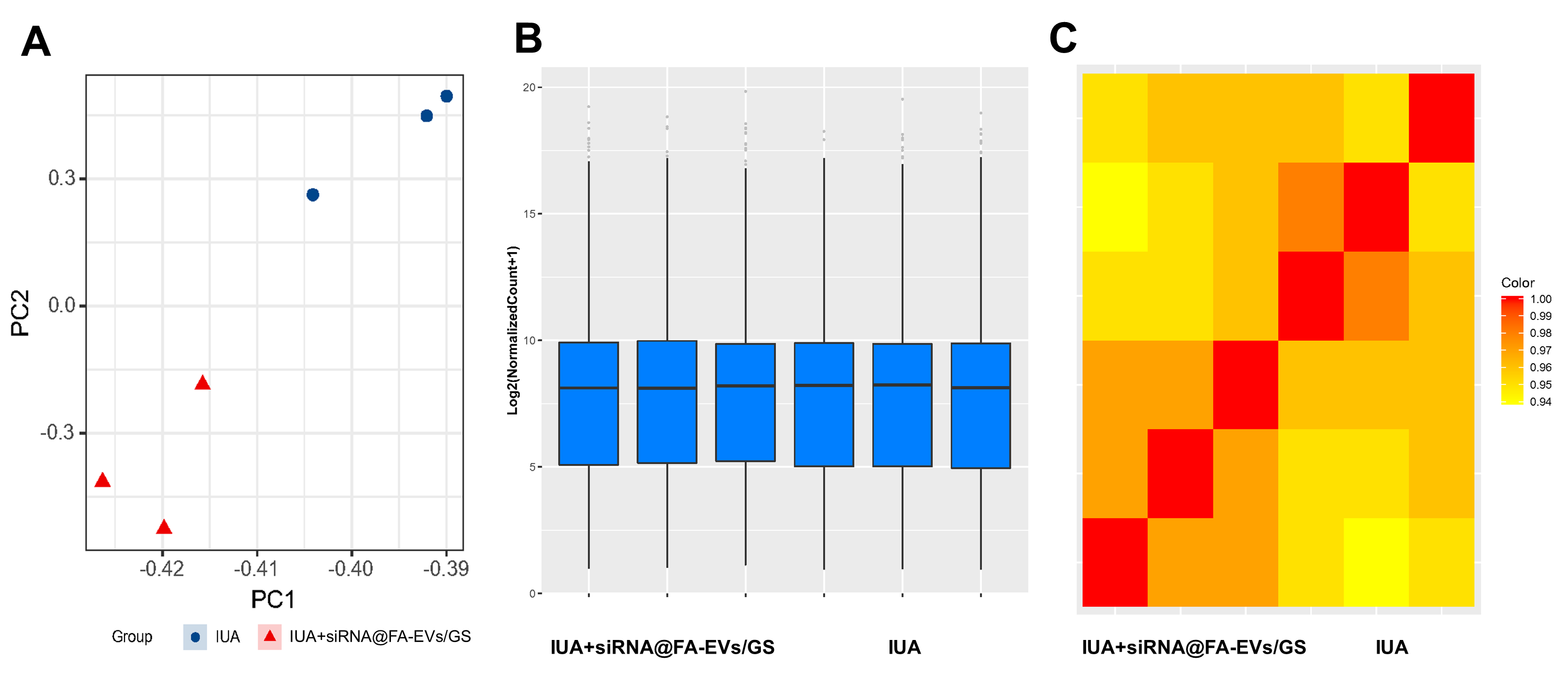


**Figure S20.** A) Principal component analysis (PCA) plot; B) boxplot of normalization data of each group; C) sample-to-sample distance heat maps between IUA and IUA+siRNA@FA-EVs/GS group.


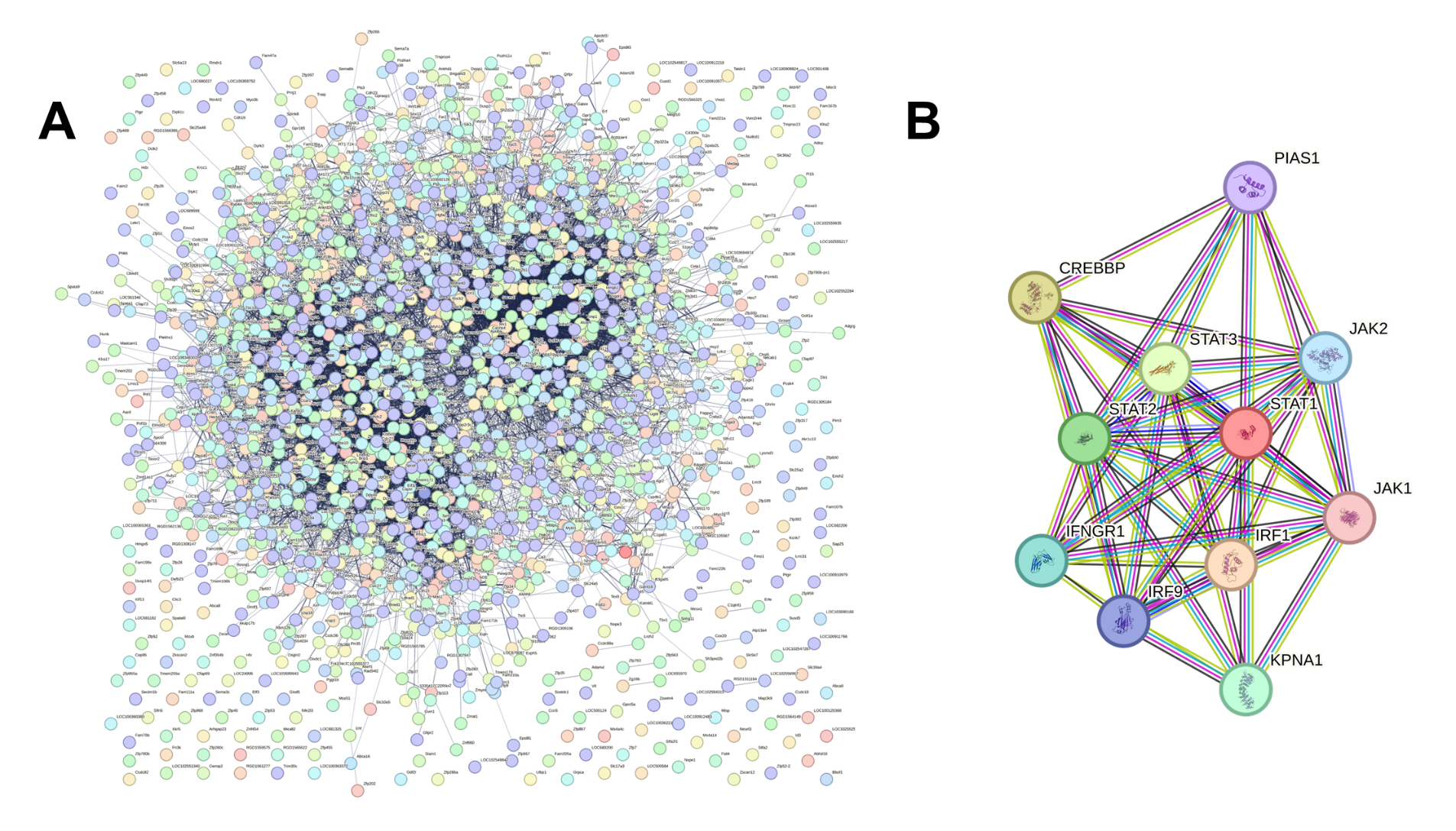


**Figure S21**. A) DEGs between IUA and IUA+siRNA@FA-EVs/GS groups were visualized in the PPI network. B) DEGs related to STAT1 between IUA and IUA+siRNA@FA-EVs/GS groups were visualized in the PPI network.


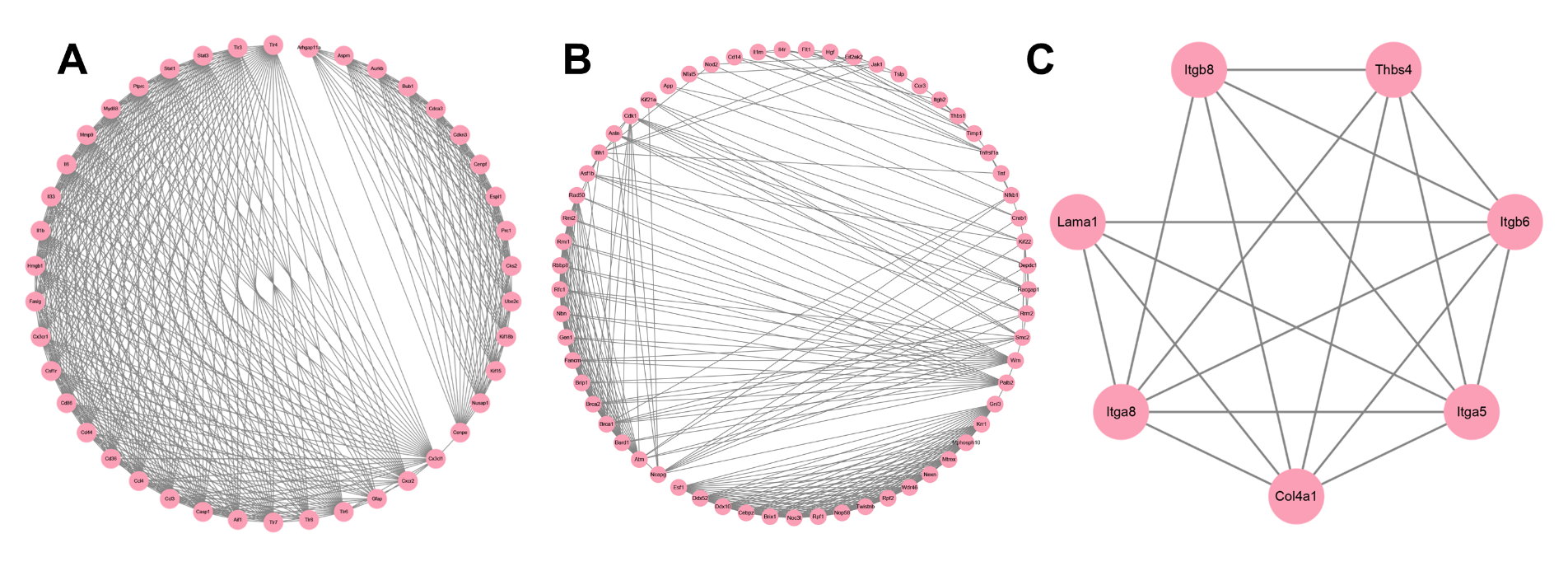


**Figure S22.** A-C) Module analysis using MCODE.


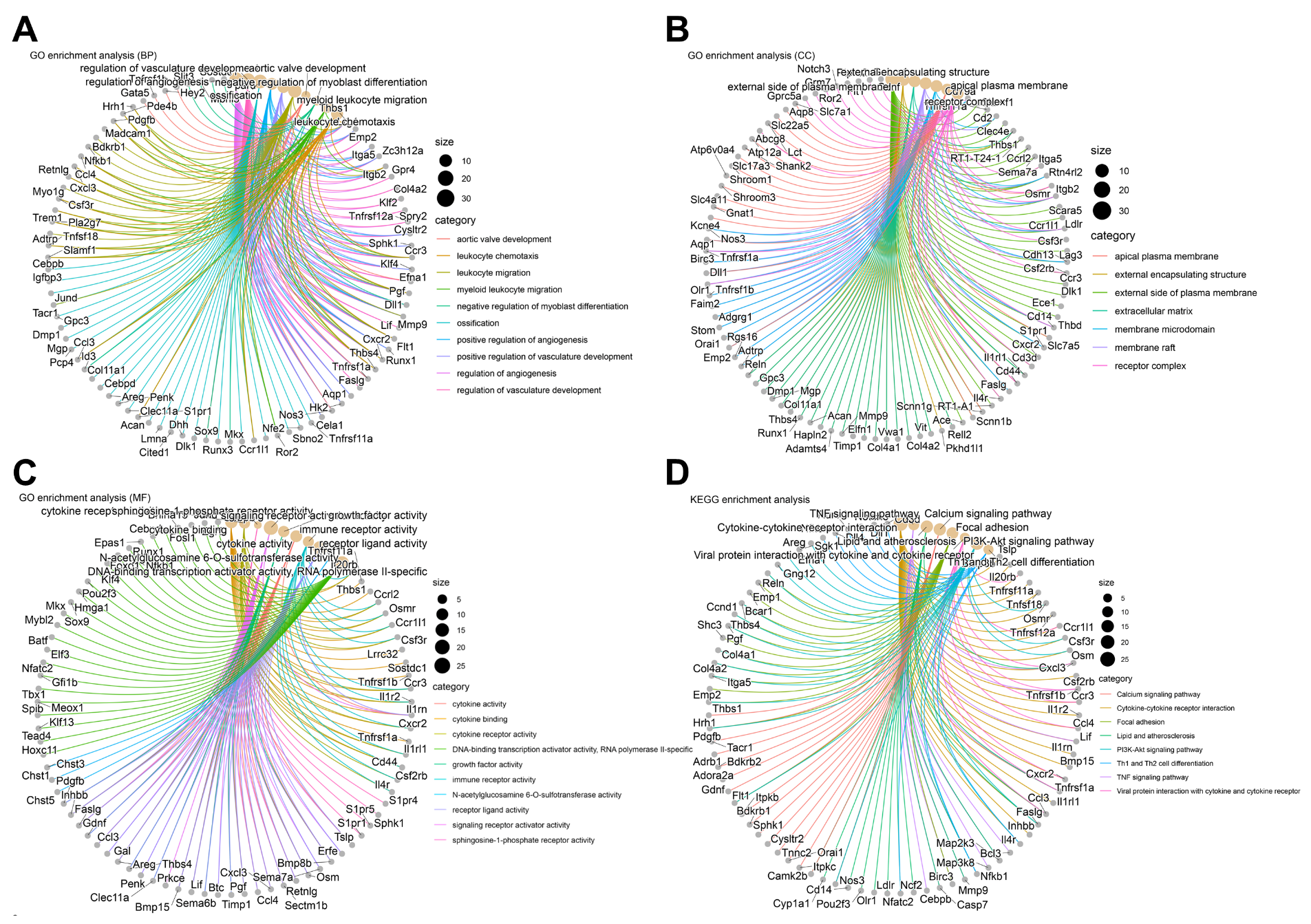


**Figure S23.** KEGG and GO analysis of upregulated DEGs between IUA and IUA+siRNA@FA-EVs/GS groups, including A) BP, B) CC, C) MF and D) KEGG.


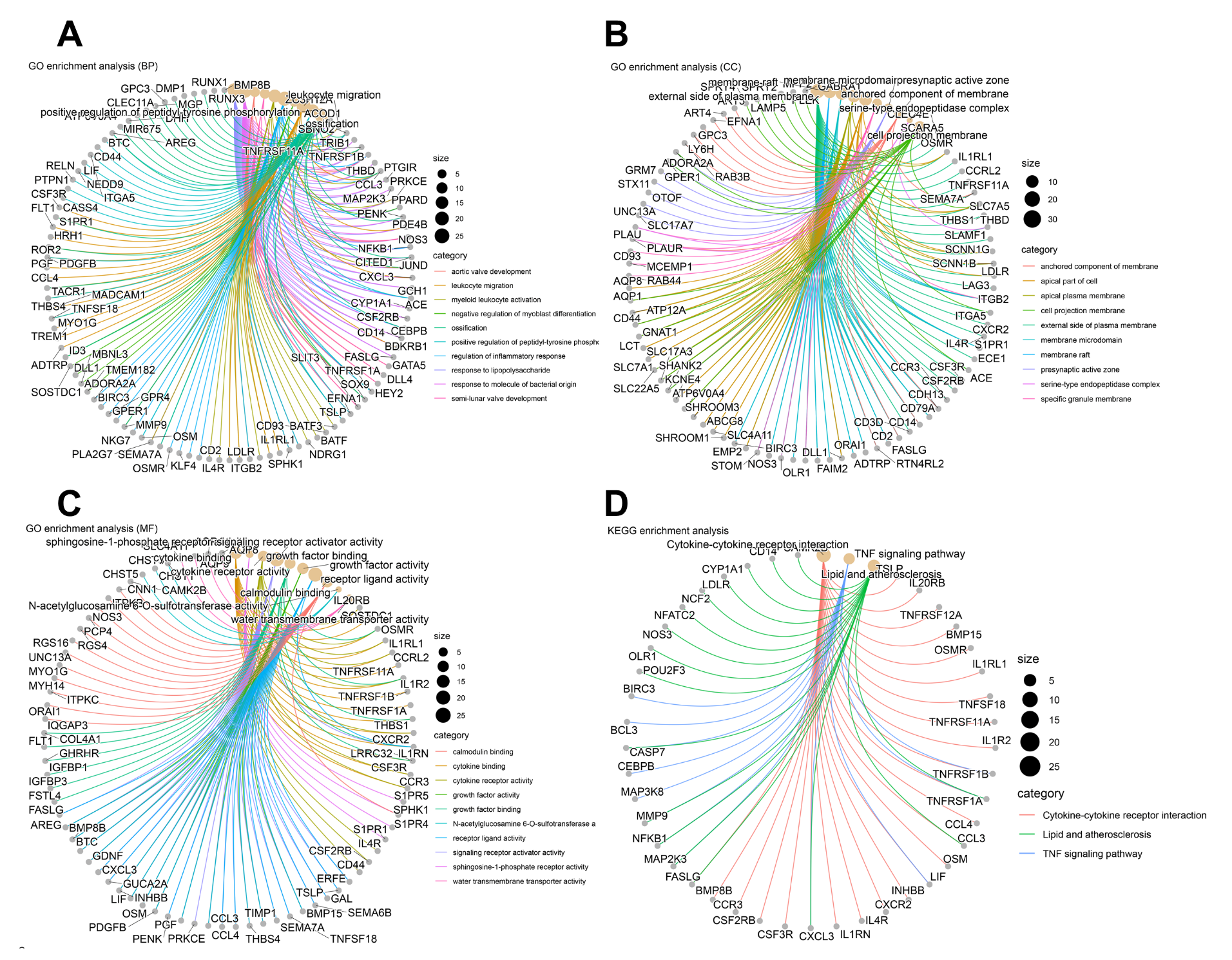


**Figure S24.** KEGG and GO analysis of downregulated DEGs between IUA and IUA+siRNA@FA-EVs/GS groups, including A) BP, B) CC, C) MF and D) KEGG.


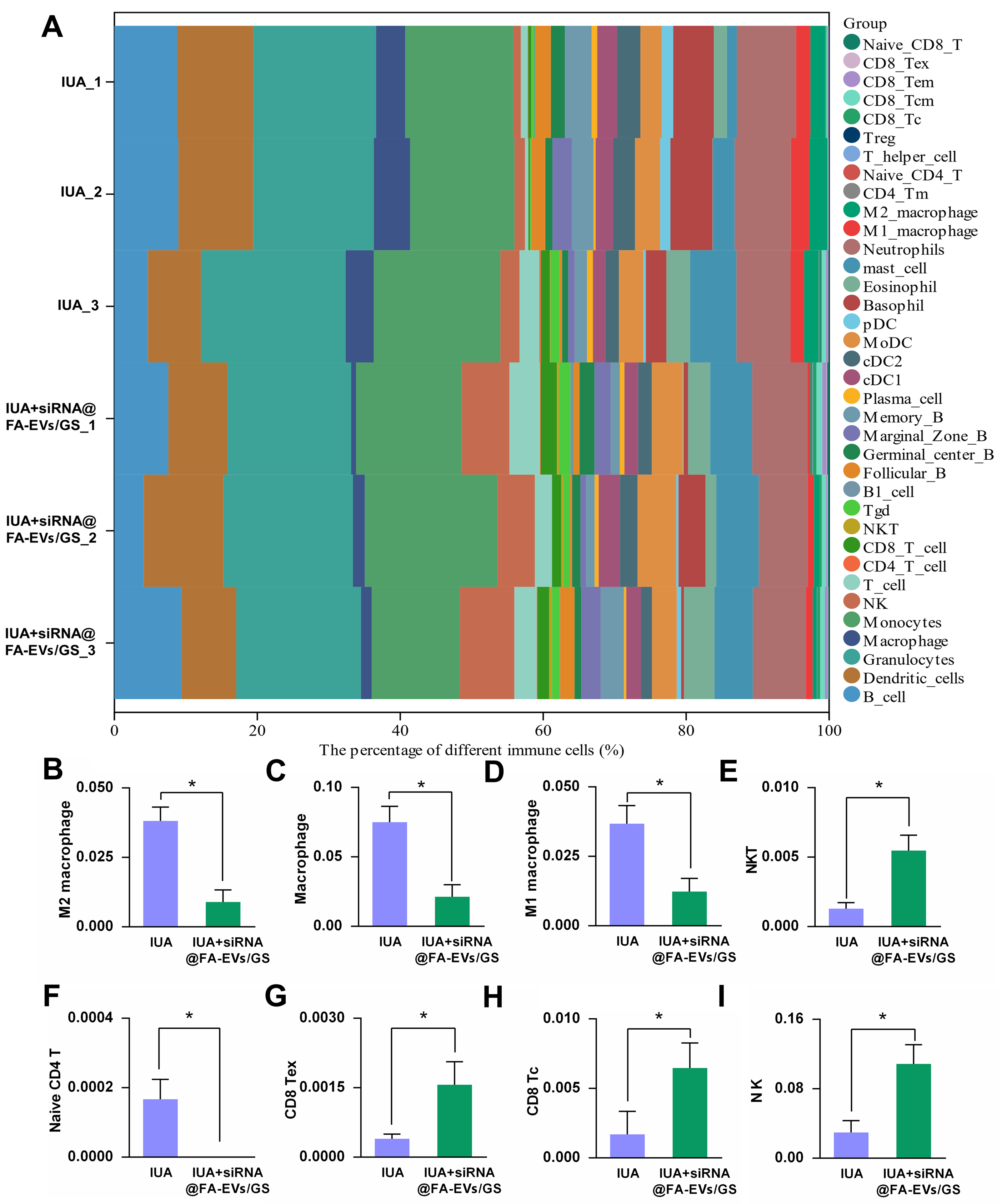


**Figure S25.** A-I) Immune infiltration analysis and proportion of various immune cells. Data are presented as mean ± SD and analyzed by unpaired two-tailed t-test. ^*^p < 0.05 compared with IUA group (n = 3).

**Table S1.** The sequences of STAT1-siRNA candidates.

|  |  | Sequence |
| --- | --- | --- |
| siRNA1 |  | Sense Sequence:  UGACCUGGAGGUCUUUGUUCCCUUU  Antisense Sequence:  AAAGGGAACAAAGACCUCCAGGUCA |
| siRNA2 |  | Sense Sequence:  ACCUGGAGGUCUUUGUUCCCUUUCA  Antisense Sequence:  UGAAAGGGAACAAAGACCUCCAGGU |
| siRNA 3 |  | Sense Sequence:  CCUGGAGGUCUUUGUUCCCUUUCAG  Antisense Sequence:  CUGAAAGGGAACAAAGACCUCCAGG |

**Table S2.** The sequences of RT-PCR primers

| Gene symbol |  | Species |  | Forward primer |  | Reverse primer |
| --- | --- | --- | --- | --- | --- | --- |
| STAT1 |  | Mouse |  | TCACAGTGGTTCGAGCTTCAG |  | CGAGACATCATAGGCAGCGTG |
| CD86 |  | Mouse |  | TCAATGGGACTGCATATCTGCC |  | GCCAAAATACTACCAGCTCACT |
| IL-1β |  | Mouse |  | GAAATGCCACCTTTTGACAGTG |  | TGGATGCTCTCATCAGGACAG |
| TNF-α |  | Mouse |  | CAGGCGGTGCCTATGTCTC |  | CGATCACCCCGAAGTTCAGTAG |
| α-SMA |  | Mouse |  | CCCAGACATCAGGGAGTAATGG |  | TCTATCGGATACTTCAGCGTCA |
| Col III |  | Mouse |  | CTGTAACATGGAAACTGGGGAAA |  | CCATAGCTGAACTGAAAACCACC |
| FN1 |  | Mouse |  | ATGTGGACCCCTCCTGATAGT |  | GCCCAGTGATTTCAGCAAAGG |
| CD86 |  | Rat |  | AAACATAAGCCCGAGTGAGC |  | TGTGAAGTCGTAGAGCCTGGT |
| α-SMA |  | Rat |  | TTCGTGACTACTGCTGAGCG |  | CTGTCAGCAATGCCTGGGTA |
| Col III |  | Rat |  | CCTGAACTCAAGAGCGGAGAA |  | CATGGCCTTGCGTGTTTGAT |
| FN1 |  | Rat |  | GGATCCCCTCCCAGAGAAGT |  | GGGTGTGGAAGGGTAACCAG |
| IL-1β |  | Rat |  | GGGCCTCAAGGGGAAGAATC |  | ATGTCCCGACCATTGCTGTT |
| TNF-α |  | Rat |  | GGCTTTCGGAACTCACTGGA |  | CCCGTAGGGCGATTACAGTC |
